# Polygenic strategies for host-specific and general virulence of *Botrytis cinerea* across diverse eudicot hosts

**DOI:** 10.1093/genetics/iyaf079

**Published:** 2025-06-09

**Authors:** Céline Caseys, Daniel J Kliebenstein

**Affiliations:** Department of Plant Sciences, University of California, Davis, CA 95616, USA; Department of Plant Sciences, University of California, Davis, CA 95616, USA

**Keywords:** fungal virulence, host range, host–pathogen interaction, *Botrytis cinerea*, crops, GWAS, quantitative genetics, generalist pathogen, fungi

## Abstract

Diverse qualitative and quantitative genetic architectures can successfully enable fungal virulence and host range. To model the quantitative genetic architecture of a generalist pathogen with an extensive host range, we conducted a genome-wide association study (GWAS) of the lesion area of *Botrytis cinerea* across 8 hosts. This revealed that it was possible to partition the virulence, as defined by the lesion area, common across all hosts from host-specific virulence. All traits showed that a large proportion of the *Botrytis* genome likely contributes to fungal lesion development on leaves with small effect sizes. The candidate genes are evenly spread across the core chromosomes with no indication of bipartite genomic architecture. The GWAS-identified polymorphisms and genes show that *B. cinerea* relies on genetic variants across hundreds of genes for growing on diverse hosts, with most genes influencing relatively few hosts. When pathogen genes were associated with multiple hosts, they were associated with unrelated rather than related host species. Comparative genomics further suggested that the GWAS-identified genes are largely syntenic with other specialist Botrytis species and not unique to *B. cinerea*. Overall, as shown in *Arabidopsis thaliana*, *B. cinerea*'s generalist behavior is derived from the sum of the genome-wide genetic variation acting within gene networks that differentially coordinate the interaction with diverse hosts.

## Introduction

Plant pathogens are major threats to global food security as they cause significant yield losses (Strange and Scott 2005; Singh *et al*. 2023a). However, most plant–microbe interactions do not result in disease, either due to the success of the plant defenses or the microbes’ lack of virulence. Those defense/virulence molecular strategies are encoded in the host and microbes genomes and the genetic variation in plants interacts with the genetic diversity within pathogens (Laine *et al*. 2011; Barrett *et al*. 2015). It is critical to understand how the genetic variation in both organisms leads to disease and the pathogen's ability to cause disease in single to multiple hosts, also known as host range (Barrett *et al*. 2009; Morris and Moury 2019).

For some plant–pathogen interactions, the genetics determining the interaction relies on a few genetic variants of large effect creating qualitative variation in the virulence outcome (Dong *et al*. 2015; Möller and Stukenbrock 2017). These large-effect genes can be present on accessory chromosomes such as in *Alternaria alternata*, close to repetitive elements such as in *Magnaporthe oryzae*, or close to recombination hotspots such as in *Melampsora lini* (Li *et al*. 2020). Often clusters of these genes are associated together and evolve more rapidly than the rest of the genome leading to the appearance of a 2-speed genome (Dong *et al*. 2015; Torres *et al*. 2020). In other species, such as *Blumeria graminis*, host specialization genes do not appear to cluster but are boosted by a high duplication rate and a dynamic secretome (Frantzeskakis *et al*. 2018). Horizontal transfer of those effector genes or accessory chromosomes between pathogen species can further influence the genetics determining host range (Mehrabi *et al*. 2011).

Beyond large-effect genes, the genetic architecture of virulence and host range can also be quantitative involving broad sets of genes (Poland *et al*. 2009), each contributing a small effect to the outcome (Liao *et al*. 2022). The broad sets of genes influencing virulence and host range in the quantitative systems tend to involve an equally diverse set of molecular mechanisms. For example, in *Zymoseptoria tritici*, quantitative pathogenicity genes are hotspots for diversifying selection (Amezrou *et al*. 2024), with heterochromatin accessibility and transposable elements (TEs) also playing an important role in creating variation (Fraser and Whitehall 2022). In *Sclerotinia sclerotiorum*, quantitative effects on virulence and host range can involve the production of distinct small RNAs (Derbyshire *et al*. 2019), transcriptional reprograming (Allan *et al*. 2019; Kusch *et al*. 2021), and alternative splicing (Ibrahim *et al*. 2021). How the quantitative architectures vary with the lifestyle (biotroph vs necrotroph) and host range of plant pathogens remains poorly understood. We used *Botrytis cinerea* to investigate how quantitative genetics influence virulence and host range in a generalist necrotroph.

*Botrytis cinerea* (Gray mold, Botrytis thereafter) is a plant pathogen that causes damage to over a thousand plant species from more than 600 genera across a wide phylogenetic spectrum, ranging from mosses to monocots and eudicots, including major crops (Elad *et al*. 2016; Singh *et al*. 2023b). Botrytis threatens food security both preharvest and postharvest, with the ability to attack diverse hosts and organs within each host such as the leaf, flower, fruits, and stem (Dean *et al*. 2012). The mechanism(s) enabling *B. cinerea* to be such a successful generalist are not clear given that other Botrytis species tend to be specialists (Navaud *et al*. 2018; Valero-Jiménez *et al*. 2019; Valero-Jiménez *et al*. 2020; Garfinkel 2021). Thus, this system has the potential to provide insight into how generalist plant pathogens may evolve and the mechanisms involved.

As a necrotroph, Botrytis infections can lead to the formation of lesions associated with host cell death and the fungal development in the decaying tissues (Veloso and van Kan 2018; Bi *et al*. 2023). The genetic architecture of Botrytis infections and lesion development is highly quantitative with over a hundred genes having a validated role in modulating virulence and potentially host range (Williamson *et al*. 2007; Nakajima and Akutsu 2013; Mbengue *et al*. 2016; Veloso and van Kan 2018; Cheung *et al*. 2020; Bi *et al*. 2023; Singh *et al*. 2023b). These genes include but are not limited to signaling genes in molecular pathways (Doehlemann *et al*. 2006; Tudzynski and Gronover 2007) such as cAMP (Klimpel *et al*. 2002; Schumacher *et al*. 2008), G proteins (Gronover *et al*. 2004), calcium-dependent (Harren *et al*. 2012; Kilani *et al*. 2020), and MAPK cascades (Rui and Hahn 2007; Schamber *et al*. 2010; Heller *et al*. 2012) that are historically associated with virulence in fungi with other lifestyles (e.g. biotrophs).

Mechanistically, Botrytis relies on diverse output mechanisms to interact with the host, including through small RNAs that interfere with the host's resistance mechanisms (Weiberg and Jin 2015). Other major parts of the Botrytis virulence toolbox are toxins (such as botrydial) (da Silva Ripardo-Filho *et al*. 2023), cell wall degrading enzymes, and cell death-inducing proteins (Mbengue *et al*. 2016; Bi *et al*. 2023). Further contributing to the host cell death are genes modulating redox, pH, and the active generation of reactive oxygen species (Heller and Tudzynski 2011; Newman and Derbyshire 2020). In addition to the above offensive mechanisms, Botrytis also has numerous and diverse defensive mechanisms, such as efflux via transmembrane transporters, to resist the various phytochemical defenses produced by the broad range of host plants (Stefanato *et al*. 2009; Pedras *et al*. 2011; Westrick *et al*. 2021; Kuroyanagi *et al*. 2022; Bulasag *et al*. 2023). How this diversity of molecular mechanisms and potentially other unknown mechanisms contribute to influencing virulence across the broad-host range of *B. cinerea* is presently unclear.

With over a thousand plant hosts (Elad *et al*. 2016; Singh *et al*. 2023b), it is imperative to begin modeling how many and what type of Botrytis genes contribute to virulence on a single host vs multiple hosts. Is a broad-host range due to host-specific genes or genes functioning across diverse hosts using host-specific alleles? For example, different alleles of Polygalacturonase 1 (PG1) appear optimized to degrade different pectin structures across host families (Rowe and Kliebenstein 2007). However, the relative contribution of host-specific genes or host-specific alleles remains to be assessed. Furthermore, identifying the genes influencing Botrytis virulence across diverse host plants will help understand the origins of extreme polyphagy within the Botrytis genus (Navaud *et al*. 2018; Mercier *et al*. 2019). Such polyphagy could result in the evolution of novel genes or retention/collection of host-specific genes found in other Botrytis species.

**Fig. 1.**
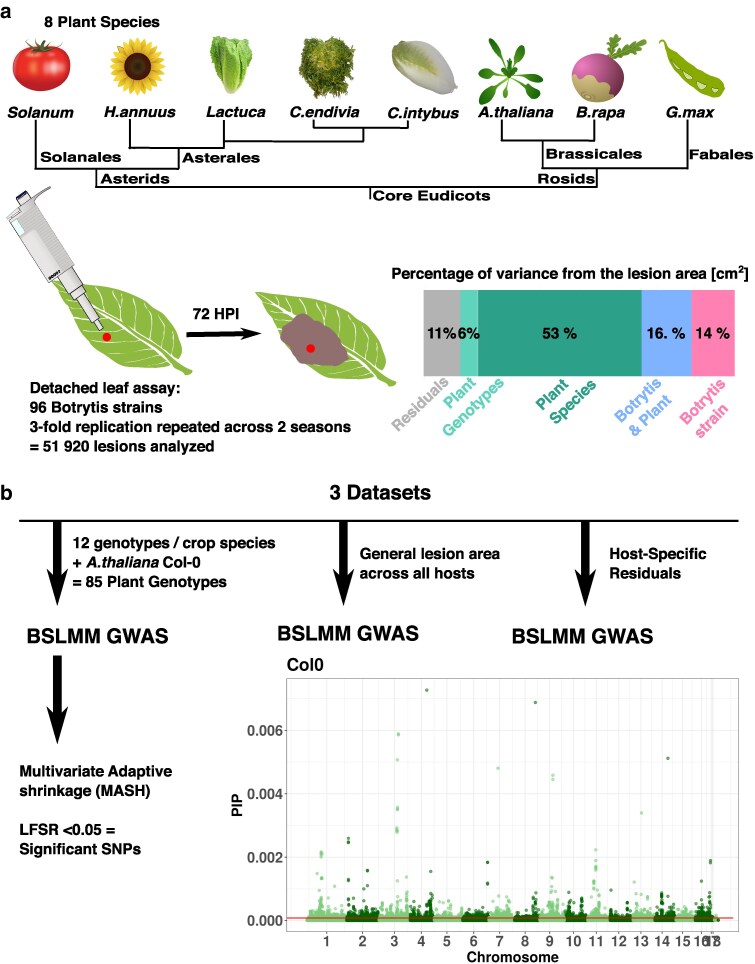
a) Overview of the phenotypic dataset previously published in Caseys *et al*. (2021). To test the virulence of *B. cinerea* across plant species, 8 crops were selected to represent a range of phylogenetic distances and domestication histories and were infected with 96 strains of the fungal pathogen. From these replicated detached leaf assays, the lesion area at 72 h postinoculation was calculated by image analysis and modeled to estimate the contributions of the plants and fungal strains (Caseys *et al*. 2021). b) Experimental design and data analysis pipeline for this study. Three datasets based on the phenotypic data from Caseys *et al*. (2021) were generated and analyzed with BSLMM: (1) the lesion area across the 85 genotypes, (2) the general lesion area across all hosts, and (3) the host-specific residuals. The 85 genotypes dataset was subsequently analyzed for multivariate inferences. Each specific aspect of the experiment is covered in detail in the Materials and methods.

To begin querying how the host range and virulence are determined in Botrytis, we used previous measurements of the lesion area caused by a population of 96 diverse Botrytis strains across an array of eudicots (Fig. 1a) including tomato, sunflower, lettuce, chicory, endive, turnip, Arabidopsis, and soybean (Caseys *et al*. 2021). In the previous study, we used the data to focus on a phenotypic description of the disease outcome, lesion area. As summarized in Fig. 1a, lesion area reflects the interaction of each Botrytis strain with each plant host, with both the plant resistance and fungal virulence playing a significant role (Caseys *et al*. 2021). In this study, we use the same data to explore the genetic architecture behind Botrytis’ contribution to the lesion area on each of the 8 hosts. By partitioning the phenotypic measurements between general and host-specific virulence, we begin to identify the Botrytis genes that may shape lesion development across hosts. Combining the phenotypic measurements with the genomic variation in the Botrytis population (Atwell *et al*. 2015, 2018; Soltis *et al*. 2019; Zhang *et al*. 2019; Krishnan *et al*. 2023), we mapped and analyzed the genetic architecture of host preferences across Botrytis strains using genome-wide association study (GWAS).

## Materials and methods

### Quantifying plant–pathogen interactions

A collection of 96 strains of *B. cinerea* was used to infect 85 plant genotypes across 8 different eudicot species (Fig. 1) and generate a phenotypic dataset that was described in Caseys *et al*. (2021). The Botrytis strains originated largely from California and were collected on 14 different plant hosts (Caseys *et al*. 2021). No population structure was found in the strain collection, with low genetic differentiation between strains collected in California and elsewhere (Caseys *et al*. 2021). The strain collection was therefore treated as single large population. The plant genotypes (Supplementary Table 1) cover 8 species, including *Arabidopsis thaliana,* and 7 crops. For each crop, there are 6 genotypes representing accessions either from wild or landrace origin and 6 genotypes are inbred lines or cultivars (Caseys *et al*. 2021). The crop species were selected both within the Rosids and the Asterids. For the Rosids, *Brassica rapa* (turnip) and *A. thaliana* are in the Brassicales while *Glycine max* (soybean) is in the Fabales. Within the Asterids, a range of phylogenetic distances (Fig. 1) are present. *Solanum lycopersicum* (domesticated tomato) and *Solanum pimpinellifolium* (wild tomato) are in the Solanales. Within the Asterales, 2 sister chicory species *Cichorium intybus* and *Cichorium endivia* were compared with *Lactuca sativa* (domesticated lettuce), *Lactuca serriola* (wild lettuce), and *Helianthus annuus* (sunflower).

Detached leaf assays were performed with 6-fold replication across 2 independent experiments, separated by several months, by infecting the adaxial surface of adult leaves with 4 µl drops of 50% grape juice containing 40 Botrytis spores (Caseys *et al*. 2021). The results of infections on detached leaves and whole plant typically correlate across different plant systems (Mengiste *et al*. 2003; Denby *et al*. 2004; Kirkby *et al*. 2023). To allow testing a large collection of Botrytis strains at high-throughput, the infection of detached leaves was used. As Botrytis germination is inconsistent in water and there is genetic variation in germination on single sugar sources (Clark and Lorbeer 1977), inoculation with 50% grape juice, as a blend of natural sugar and minerals, was used to maximize germination across all strains (Blakeman 1975; Benito *et al*. 1998; Denby *et al*. 2004). The lesion areas were quantified 72 h postinfection by taking pictures of lesions, counts of pixels within the lesion, and conversion into square centimeters. To account for technical errors from the inoculation process, failed lesions were identified and removed according to a statistical model described in detail in Caseys *et al*. (2021). To account for the effects of experimental factors (e.g. individual plant the leaf was collected from, the environmental effect across the 2 experiments, block effect in the experimental design), the lesion areas were analyzed with linear mixed models within each plant genotype: Lesion area ∼Strain + (1|Experiment) + (1|Block) + (1|Individual Plant). The 85 genotypes dataset contain the model-corrected least-square means estimated from this model (Caseys *et al*. 2021).

To address how strain-specific effects like average growth rate may impact Botrytis lesion area across all hosts, we estimated the least-square means of the general lesion area of each strain across the 8 eudicot species with the linear mixed model: Lesion area ∼Strain + (1|Species) + (1|Experiment) + (1|Block) + (1|Individual Plant), in which the host species were set as random factors. This approach returns the lesion size potential of each strain across all 8 hosts. This estimate was chosen because Botrytis mycelial growth rate on in vitro media shows little correlation across different artificial media and is a poor predictor of lesion area in vivo. Furthermore, lesion area when comparing across diverse Botrytis strains or host genotypes in vivo is not highly correlated with fungal biomass as measured microscopically or by transcript abundance (Corwin *et al*. 2016; Zhang *et al*. 2019). We also estimated the least-square means of the lesion area of each strain within each plant species with the linear mixed models: Lesion area ∼Strain + (1|Genotypes) + (1|Experiment) + (1|Block) + (1|Individual Plant) in which the 12 host genotypes were set as random factors. Finally, we calculated an estimate of how host-specific interactions differ from the general lesion potential for each strain by extracting the residuals from the linear modeling of the host-specific lesion formation against the general lesion formation (Supplementary Fig. 1). These host-specific residuals represent the strain-specific deviations in lesion development from their general lesion estimates on a specific host.

### Genome-wide association studies

To map the genetic variation in Botrytis influencing lesion area, we used 3 trait sets: (1) the lesion area on the 85 plant genotypes, (2) the general lesion area across all hosts, and (3) the host-specific residuals. These 3 trait sets were then used for GWAS (Fig. 1). For the genotyping information, we used a previously published set of 271,749 single nucleotide polymorphisms (SNPs) at a minor allele frequency (MAF) of 0.2 with <10% missing data mapped (Atwell *et al*. 2015, 2018; Soltis *et al*. 2019) to the B05.10 genome ASM83294v1 assembly (Van Kan *et al*. 2017).

No evidence of host specialization or population structure was detected among the 96 strains of this haploid fungus (Atwell *et al*. 2018; Caseys *et al*. 2021). Given the quantitative nature of Botrytis virulence (Soltis *et al*. 2019; Caseys *et al*. 2021), a Bayesian sparse linear mixed model (BSLMM) using Markov chain Monte Carlo algorithm implemented in Genome-wide Efficient Mixed Model Association was run for lesion area on each plant genotype (Zhou and Stephens 2012). BSLMM was chosen because it concurrently models 2 effect size distributions: a polygenic architecture made of loci of small effect sizes and an oligogenic architecture allowing for some loci of modest to larger effect sizes (Zhou *et al*. 2013). A standardized relatedness matrix was included in the model to account for genetic similarity and any population structure. For each trait, 20 independent runs with 500,000 burn-in and 5,000,000 iterations with recording every 10 steps were performed. For these models, we constrained the parameter *h*, an estimate of the narrow-sense heritability, between 0.1 < *h* < 0.9. All studies to date (Zhang *et al*. 2017; Soltis *et al*. 2019; Caseys *et al*. 2021) have shown heritability rates within this range for Botrytis virulence.

BSLMM outputs both SNP estimates of effect size and significance, estimated as the posterior inclusion probability (PIP). For each SNP, the median of the effect size and PIP distribution of the 20 runs was used for subsequent analyses. To estimate the null distribution of the PIP, 10 random permutations were performed for each host species revealing a common trend across species (Supplementary Fig. 2). From the random permutation, it was estimated that PIP values larger than 1.7 ×10^−4^ are equivalent to a 5% chance of false positives. The BSLMM results were then filtered using this 5% chance of false-positive threshold.

For the 85 genotypes dataset, we implemented a multivariate approach to the BSLMM using multivariate adaptive shrinkage (MASH) (Urbut *et al*. 2018) to incorporate the phenotypic information potentially provided by lesion area measured on up to 12 plant genotypes per host species. MASH analyzes the combined effects and standard errors of GWAS-associated SNPs across the phenotypes while accounting for arbitrary correlations among conditions. This multivariate approach did not result in significant results when combining the GWAS across all the host species indicating the absence of universally significant SNPs. MASH models were then applied to each host using all the independent genotypes per host and covariance matrices and local false discovery rate (LFSR) posterior summaries were extracted. Those LFSRs are analogous to the false discovery rate. SNPs with LFSR <0.05 were considered significant for the given host genotype and significances consolidated within each species.

To test for relationships in the SNP significances across the host genotypes, we conducted a hierarchical clustering across the 85 genotypes using the “complete” agglomeration method. This method calculates the maximum Euclidean distance between clusters before merging. The significance of the branches was assessed with the r package pvclust over 20,000 bootstraps. Branches were declared significant if the approximately unbiased *P*-values (AU) estimated using bootstraps were larger than 95%.

To obtain a genome-wide view of the distribution of the significant SNPs and their local effect, an effect size sliding window analysis was performed. Given the level of linkage disequilibrium (Atwell *et al*. 2018; Mercier *et al*. 2021) within Botrytis and the potential for multiple significant SNPs within genes, we used a sliding window of 1 kb, with steps of 500 bp. Within each window, we summed the local effect size estimates for each SNP within the window.

### Gene-level analyses

Because the average haplotype diversity and linkage disequilibrium along the Botrytis genome provides approximately gene-level resolution for mapping (Atwell *et al*. 2018; Mercier *et al*. 2021), we consolidated the significant SNPs identified into their associated genes for further analyses. The gene was defined as inclusive of CDS, introns, and untranslated regions (UTR). To take into account regulatory regions, SNPs within 500 bp of the start or end of the gene were also included. This is standard practice in GWAS as studies have shown that causal SNPs are often in noncoding regions (Brodie *et al*. 2016). If an SNP was in an area with presence of a TE in the B05.10 reference genome, it was annotated as such. TEs in the B05.10 genome were annotated based on REPET TEdenovo (Porquier *et al.* 2016). Genes were annotated with gene names, genomic location, protein features, and function prediction as present on fungidb.org (Basenko *et al.* 2018) in January 2024. ncRNAs were annotated with tRNAscan-SE (Chan and Lowe 2019). Further annotations were added manually for genes part of the surfactome (Escobar-Nino *et al.* 2021) and secretome (González-Fernández *et al*. 2015).

To reveal how the GWAS-identified Botrytis genes were shared across host plant species, a Venn diagram with 7 species (excluding *A. thaliana,* which has only 1 genotype) was drawn with the R library “Venn.” This representation with 7 species is the most complete option available. Furthermore, hierarchical clustering of the genes across hosts was run as the SNP analysis described above.

To estimate the proportion of the genes that might have recently evolved within *B. cinerea*, we performed comparative genomics analysis with 7 Botrytis species. *Botrytis cinerea* B05.10 genes orthology to *Botrytis fragariae* (Wu *et al*. 2021), *Botrytis aclada*, *Botrytis deweyae*, *Botrytis porri*, *Botrytis hyacinthi,* and *Botrytis sinoallii* (Valero-Jiménez *et al*. 2019, 2020) were called by OrthoMCL in KBase (Arkin *et al*. 2018). *Botrytis fragariae* is found in strawberry fields in Germany and South East United States (Rupp *et al*. 2017). *Botrytis deweyae* is an endophytic facultative necrotroph, found on 1 plant species (polyphagy index = 1). All other species are necrotrophs infecting monocots infecting 3–9 host species and polyphagy index between 1.4 and 3.2 (Mercier *et al*. 2019).

To estimate putative mechanistic connections among the GWAS-identified genes, gene coexpression networks were drawn using 16 h postinoculation transcriptomes measured from these same Botrytis strains infected on *A. thaliana* Col-0 leaves (Zhang *et al*. 2017, 2019). In the networks, vertices connect transcripts with a Spearman's rank correlation of *ρ* > 0.75 and a Benjamini–Hochberg adjusted *P*-value < 0.05. Networks were constructed with the R package “network” and plotted with the gplot function of the package “sna” with vertices placed according to the Fruchterman–Reingold algorithm.

## Results

### The lesion area as a measure of Botrytis virulence

A detached leaf assay measuring the disease outcome of a population of Botrytis strains on diverse eudicot hosts was conducted in a previous study (Caseys *et al*. 2021). All experiments were run in a laboratory setting and independently replicated twice. This replication allows a focus on the genetic effects that are more likely to be stable. The lesion area associated with Botrytis necrotrophic behavior on leaves is genetically heritable and variable across the 85 plant genotypes from 8 plant species (Supplementary Fig. 3a). The percentage of total variance in the lesion area explained by genetic variation between Botrytis strains ranged from 21% (*A. thaliana*) to 51% (*C. endivia*) (Supplementary Fig. 3a). As the range of lesion area for each host genotype across the 96 Botrytis strains is wide (Supplementary Fig. 3b), the lesion area was used as a phenotype to conduct a GWAS and reveal the genetic architecture of Botrytis’ interactions with diverse host plants. We also utilized the lesion area across all 85 genotypes to develop 2 additional traits that estimate general (lesion genetic variation common across all hosts) and host-specific lesion effects. These 2 lesion estimates vary in their degree of correlation across host species. Low correlations in *C. endivia* (*R*^2^ = 0.31, Supplementary Fig. 1d) and *Solanum* (*R*^2^ = 0.37, Supplementary Fig. 1a) indicate that Botrytis strains tend to rely on host-specific mechanisms to develop lesions on those hosts. High correlations in *H. annuus* (*R*^2^ = 0.76, Supplementary Fig. 1b) and *B. rapa* (*R*^2^ = 0.71, Supplementary Fig. 1f) indicate that Botrytis strains tend to rely on their general mechanisms when interacting with those hosts. As such, the distance of each point to the linear regression, the host-specific residuals, is an indication of how Botrytis strains can specifically adjust to the hosts. The general lesion area and the host-specific residuals were further used alongside the 85 genotypes dataset for GWAS.

### Small effect sizes determine the lesion area across hosts

Given previous work on Arabidopsis (Zhang *et al*. 2019; Krishnan *et al*. 2023), tomato (Soltis *et al*. 2019), and genome scans (Mercier *et al*. 2021), we anticipated predominantly small effect loci with occasional loci having moderate effect sizes. To match the GWAS model to this architecture, we used BSLMM, which can estimate genome-wide small effects (a polygenic architecture modeled by linear mixed model) and additional moderate effects (an oligogenic architecture modeled by Bayesian regression model). Using the resulting data, we first quantified the genetic architecture of the lesion area across all the host genotypes with 4 BSLMM hyper-parameters: the proportion of heritability explained by the Bayesian regression model (rho), the percentage of variance explained by both models (PVE), the number of variants with larger effect (gamma), and the proportion of PVE explained by variants with larger effects (PGE).

This revealed that the genetic architecture of the lesion area across all plant genotypes was quantitative and complex. The quantitative genetic architecture of Botrytis interactions was similar across all 3 datasets regardless if it was measured using lesion area on the 85 genotypes (Supplementary Fig. 4), general lesion area across all hosts (Supplementary Fig. 5), or the host-specific residuals (Supplementary Fig. 5). Using BSLMM, it is possible to provide an empirical assessment of the genetic architecture by estimating a rho (*ρ*) parameter that can vary from polygenic (*ρ* = 0) to oligogenic (*ρ* = 1) (Zhou *et al*. 2013). Across the 85 genotypes, there was an average of *ρ* = 0.5 with a range of *ρ* = 0.41 (*C. intybus* PI652041, Supplementary Fig. 4a) to *ρ* = 0.58 (*C. intybus* PI651945, Supplementary Fig. 4a). This suggests the genetic architecture is largely similar across all hosts and either slightly oligogenic or polygenic, with no strong model directionality and variation across genotypes of a host (Supplementary Figs. 4 and 5). The BSLMM also estimated the percentage of phenotypic variance explained by the possible genetic architectures. Considering the effect of all 271,481 SNPs in the genome explained 16% (*L. sativa* LJ10335) to 53% (*C. intybus* PI652021) of the phenotypic variance (Supplementary Fig. 4b) across the 96 Botrytis strains. This range of percentage of phenotypic variance is likely due to variations in the influence of plant genetics across the species (Supplementary Fig. 3b). When modeling for moderate effects, the oligogenic model consistently found 10 to 15 SNPs (Supplementary Figs. 4c and 5c) influencing virulence in each genotype.

### Significantly associated SNPs are spread across the genome

**Fig. 2.**
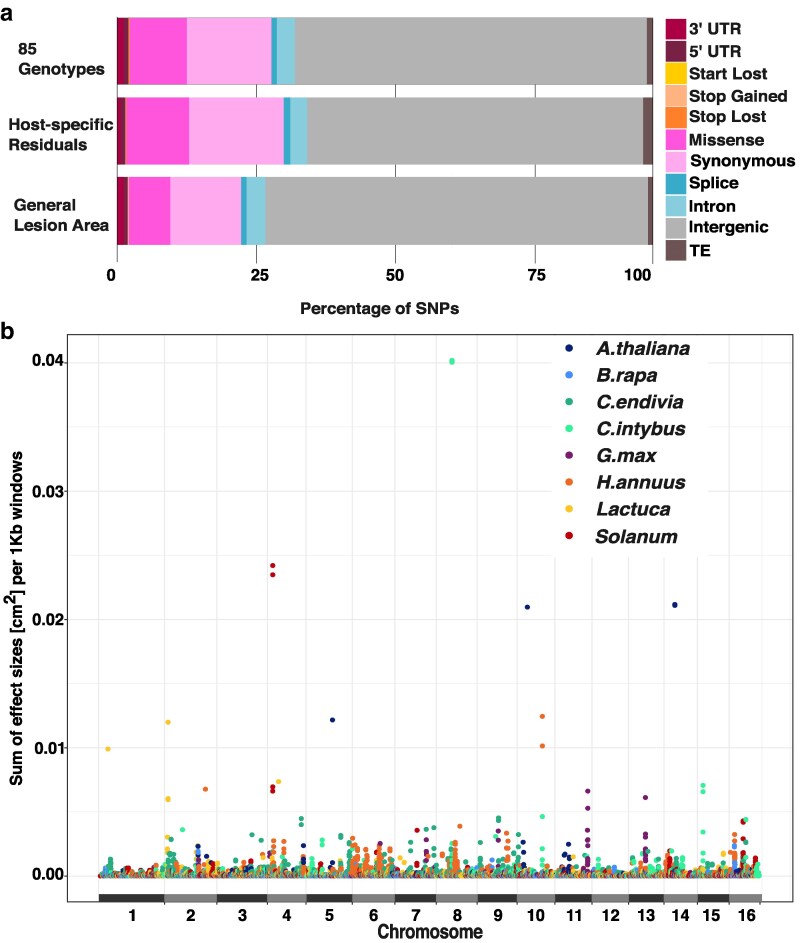
SNPs significantly associated with the lesion area are spread across genomic features and the 16 core chromosomes. a) Percentage of SNPs in genomic features and their functional effects defined as being within UTRs, coding sequences, intron, intergenic region, or within TE in the B05.10 reference genome. b) Manhattan plot of the effect size of SNPs significantly associated (LFSR < 0.05) with lesion area (cm^2^) across the 85 genotypes. The effect sizes are summed within 1 kb window. The colors indicate the host with the maximal effect size for each window.

Across all 85 genotypes, 27,068 SNPs were significantly (LFSR < 0.05) associated with the lesion area (Supplementary Table 2). The mapping of the general lesion area across all hosts revealed 1,197 SNPs (Supplementary Table 3) while the host-specific residuals identified 3,484 SNPs (Supplementary Table 4). The distribution of the SNPs across the genomic features is proportionally similar across the 3 different datasets, with SNPs largely located in the intergenic regions (62–71%, Fig. 2a). In RNA-coding regions, 8–16% of SNPs are changing the amino acid (missense) while 11–18% of SNPs conserve the amino acid (synonymous). To test if genomic features (3′UTR, 5′UTR, CDS, Intergenic, Intron) identified in the 85 genotype dataset were enriched in significant SNPs, we created a random null distribution by random selection of 27,068 SNPs that match the MAF of the significant SNPs and quantified the associated genomic features. Repeating this 100 times created permutation sets of genomic features. This showed that the significant SNPs are not enriched for gene regions (UTRs, CDS, and Introns) nor functional annotations (start/stop gain/loss, missense, and splice), but slightly enriched for the intergenic region (Supplementary Fig. 6).

To test if the SNPs were evenly distributed across the genome or potentially showed any local clustering of virulence loci (Torres *et al*. 2020), we mapped the position and effect of the significant SNPs across the Botrytis genome using 1 kb windows, which matches the average distance of linkage disequilibrium decay (Atwell *et al*. 2018). This revealed an even distribution of significant loci across the entire genome having mainly small effect sizes (Fig. 2b). SNPs identified by GWAS of the general lesion area and the host-specific residuals are also distributed across the genome with small effect sizes (Supplementary Fig. 7).

### GWAS-associated SNPs cluster by host species

At the phenotypic level, a previous analysis suggested similarities in the virulence across genotypes within a host but little phylogenetic relationship between host species (Caseys *et al*. 2021). To test how the significant SNPs may associate across host genotypes and assess if there was a signal from the phylogenetic relationships across the host species, we conducted a hierarchical clustering of all SNPs associated with lesion area across the 85 genotypes and the host-specific residuals based on their effect within a host genotype (Supplementary Figs. 8 and 9). The clustering of the SNPs confirmed the absence of a phylogenetic signal, while the 85 genotypes dataset revealed that the Botrytis causal SNPs find a common signal across genotypes within a host species. To utilize this common signal, we merged the significant SNPs across the host genotypes within a species to give a single image of the virulence architecture of Botrytis per species.

### Numerous genes influence the development of the lesion

To transition from individual SNPs and identify mechanistic signals, we used the SNP's positions in the B05.10 ASM83294V1 reference genome (Van Kan *et al*. 2017) to map significant SNPs to candidate genes. In the 85 genotypes dataset, 4,761 genes were identified (29% with functional effect variants, Supplementary Tables 2 and 5). In the host-specific residual dataset, 784 genes were identified (27% with functional effect variants, Supplementary Tables 3 and 5). For the general lesion area across all hosts, 360 genes were identified (17% with functional effect variants, Supplementary Tables 4 and 5). Using previous mechanistic studies, 72 genes identified by the GWAS have been validated for their role in Botrytis growth, development, or virulence (Supplementary Table 5). In addition to genes, 91 noncoding RNAs, including transfer RNAs, were detected (Supplementary Table 5). Such transfer RNAs have been associated with virulence in other pathogens (Böttcher *et al*. 2024; Krueger *et al*. 2024).

**Fig. 3.**
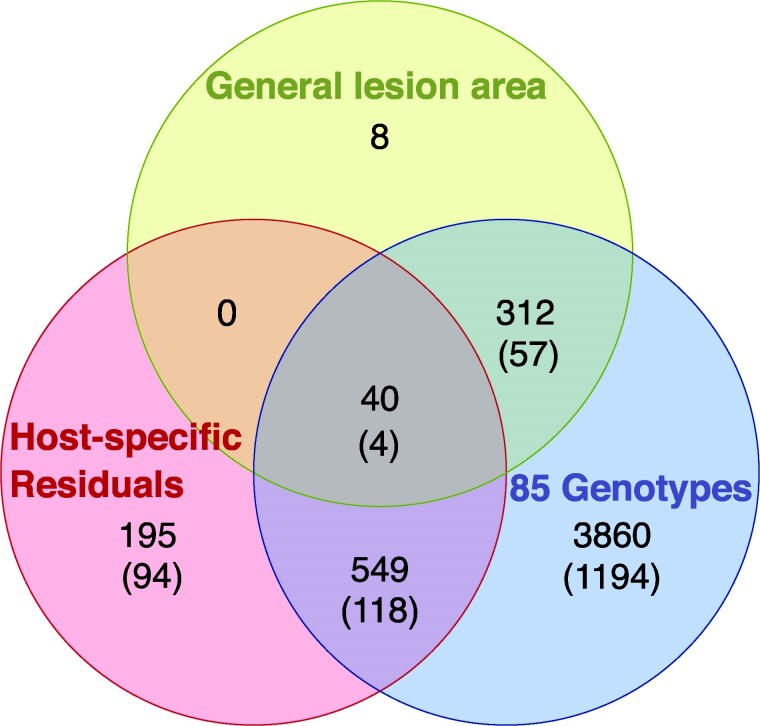
Partitioning genes influencing the general and host-specific lesion development. The Venn diagram depicts the number of Botrytis genes identified by GWAS across the 85 host genotypes, for the host-specific residuals, and for the general lesion area across all hosts. The number of genes with functional effect variants is provided in the parentheses.

To assess if the same or different genes were associated with the lesion area across the 85 genotypes, general lesion area, and host-specific residuals, we compared the 3 datasets (Fig. 3). This revealed that 352 genes (17% with functional effect variants) identified by mapping the general lesion area were also identified across the 85 genotypes. When comparing the host-specific residuals, 589 genes (20% with functional effect variants) overlapped with the 85 genotypes dataset. These numbers suggest that distinct gene sets contribute to the general lesion formation across hosts and to the host-specific processes, both contributing to the virulence of Botrytis.

### GWAS-identified genes are highly syntenic to other Botrytis species

**Fig. 4.**
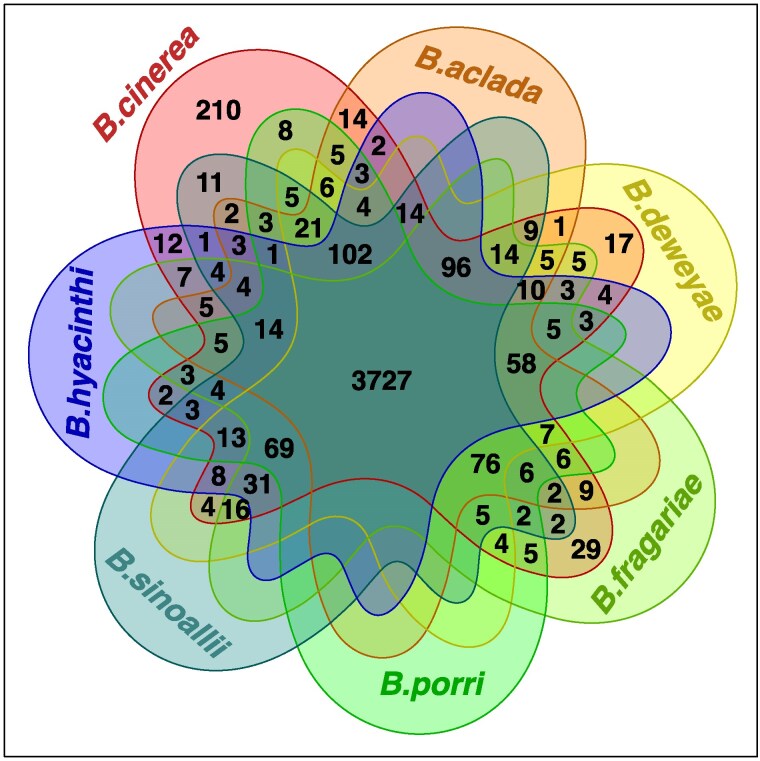
The *B. cinerea* genes detected by GWAS across the 85 genotypes are highly syntenic with other Botrytis species. Venn diagram with the count of *B. cinerea* B05.10 (clade 1, polyphagy index = 54.1) genes shared with *B. fragariae* (clade 1, polyphagy index = 1), *B. aclada* (clade 2, polyphagy index = 1.4), *B. deweyae* (clade 2, polyphagy index = 1), *B. porri* (clade 2, polyphagy index = 1.4), *B. hyacinthi* (clade 2, polyphagy index = 3.2), and *B. sinoallii* (clade 2, polyphagy index = 1.4).

Theoretically, *B. cinerea*'s ability to infect numerous hosts might result from the evolution of novel “generalist” genes or collection of host-specific genes found in other Botrytis species. To test if the genes influencing the lesion area are unique to *B. cinerea* or are present within other narrower host range Botrytis species (Valero-Jiménez *et al*. 2019, 2020), we performed comparative genomics, calling orthology groups across 7 Botrytis species from clades 1 and 2 (Garfinkel 2021). This analysis revealed that 3,727 genes identified across the 85 genotypes are fully shared across 7 Botrytis species while 794 genes are partially syntenic, being shared with 1 to 5 other Botrytis species (Fig. 4). Only 210 genes might be specific to the *B. cinerea* B05.10 reference genome. The same pattern was observed for the genes associated to general lesion area across all hosts and those associated with host-specific residuals (Supplementary Fig. 10).

To further test the significance of this pattern across the 85 genotypes, we created a null distribution of orthologs by randomly selecting a thousand sets of the *B. cinerea* orthologs (Supplementary Fig. 11a). This showed that the genes identified by GWAS are not enriched for genes specific to *B. cinerea* (Supplementary Fig. 11b). When comparing the observed (identified by GWAS) and expected distribution of genes shared with the other Botrytis species, GWAS candidate genes are less likely than by chance to be shared with *B. aclada*, *B. hyacinthi,* and *B. porri* (Supplementary Fig. 8c). It suggests that *B. cinerea*'s ability to attack a large number of hosts is primarily not through *B. cinerea* specific genes, but through a large pool of genes shared with other Botrytis species.

### The genetics behind Botrytis host range involves large gene networks

**Fig. 5.**
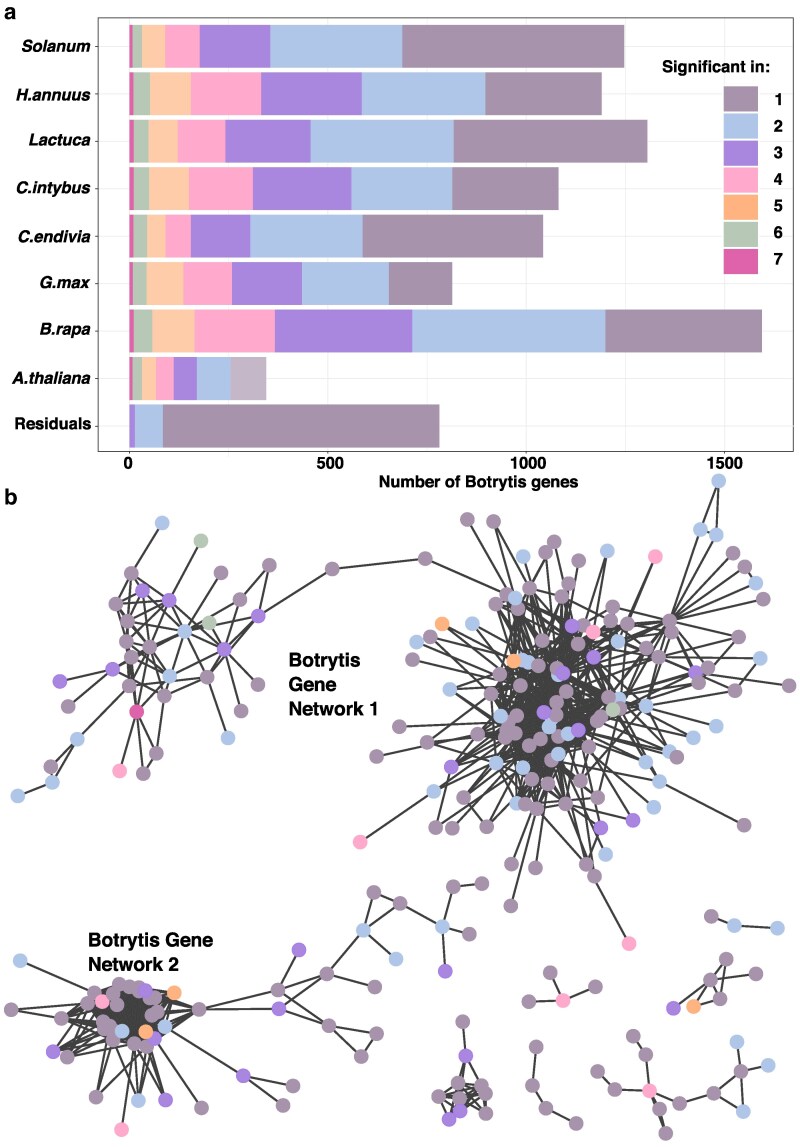
The Botrytis genes detected by GWAS are largely shared across a few hosts and coexpress within 2 large Botrytis gene networks. The gene counts are colored based on association with 1 to 7 hosts. a) Barplot of the number of Botrytis genes associated with the lesion area on each hosts and associated with the host-specific residuals. The number of associated hosts are counted within each dataset. b) Botrytis coexpression networks of the genes identified across all 85 genotypes and colored according to the number of hosts they were associated with. The Botrytis coexpression networks were generated from *A. thaliana* infection (Zhang *et al*. 2019). The vertices represent correlation *ρ* > 0.75 at Benjamini–Hochberg adjusted *P* < 0.05.

To characterize how the diverse GWAS candidate genes affect the interaction with different hosts, we counted the number of host species associated with each gene. This revealed that the genes are largely unique to 1 or 2 host species (Fig. 5a). As expected, the genes associated with host-specific residuals are largely unique to 1 or 2 hosts (Fig. 5a). Few genes identified across the 85 genotypes are associated with a majority of hosts (Fig. 5a), with 4 genes shared across 7 species, and 35 genes shared across 6 species (Supplementary Table 5). Among those 39 genes, 23 are associated with the general lesion area (Supplementary Table 5). Regarding the functions of those shared genes, 10 genes (Supplementary Table 5) contain domains such as heterokaryon incompatibility, NB-ARC, NACHT, WD40, Tetratricopeptide repeat, and Ankyrin-repeats that are associated with the regulation of cell death (Arshed *et al*. 2023; Daskalov 2023). Such genes have also been associated with virulence in *Z. tritici* using GWA approaches (Dutta *et al*. 2023).

While individual genes showed few common patterns across the diverse hosts (Fig. 5a), we proceeded to plot gene networks with potential virulence effects. We used the available Botrytis-Arabidopsis transcriptome dataset (Zhang *et al*. 2017) to plot the coexpressed candidate genes with correlation *ρ* > 0.75. This approach applied to the genes identified across the 85 genotypes revealed 2 main large networks (Fig. 5b). The Botrytis gene network 1 is composed of a majority of genes annotated as integral membrane components, active in transmembrane transport or vesicular activities. The Botrytis gene network 2 is composed of genes associated with the ribosome, translation, and protein folding activities. This analysis showed that the networks link candidate genes associated with diverse hosts. The shared genes (identified in 4 or more hosts) are spread across the networks, linked by genes identified in 1 to 3 hosts (Fig. 5b). This suggests the potential for candidate genes identified in a few hosts to influence virulence by modulating the function of key *B. cinerea* networks.

### Botrytis’ polygenic strategies do not follow the hosts’ phylogeny

**Fig. 6.**
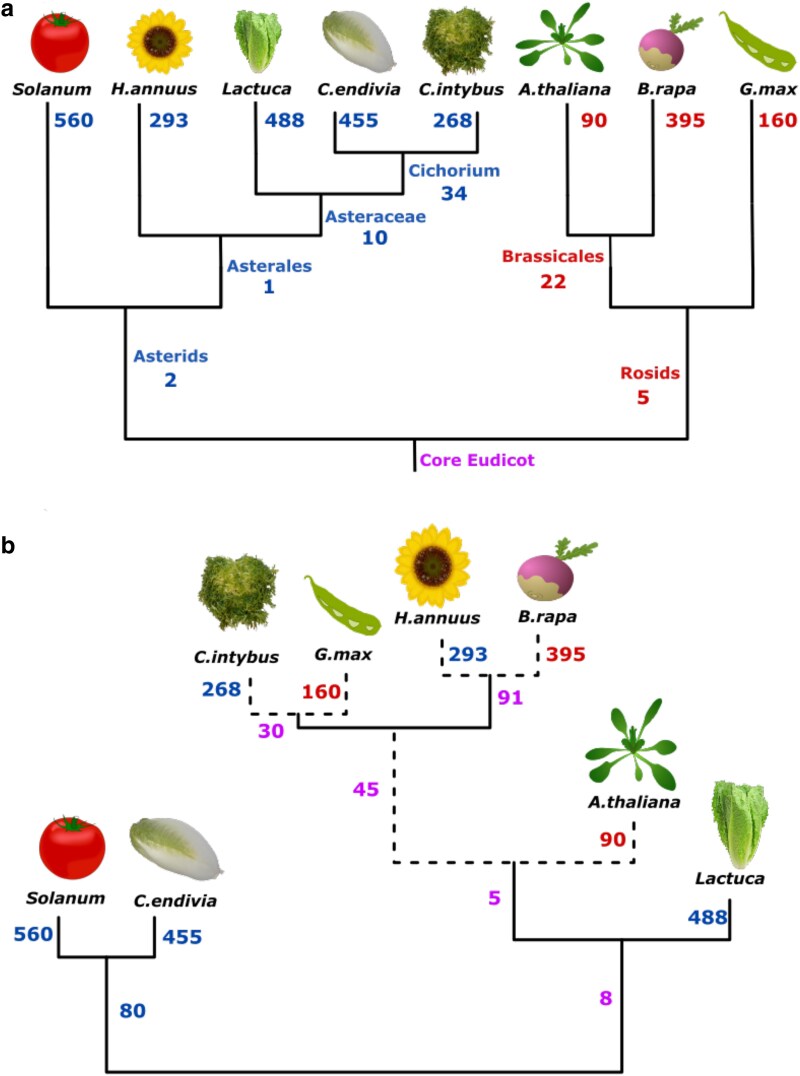
The Botrytis genes identified by GWAS across the 85 genotypes do not track the hosts’ evolutionary history. The numbers of Botrytis genes associated with lesion area in red are for rosids, in blue for asterids, and in purple for the mixed association. a) Plant-driven evolution: phylogenetic tree of the studied eudicot species with at each branch and node the number of Botrytis genes identified. b) Fungi-driven evolution: hierarchical clustering of the Botrytis genes on the different hosts. Full lines have approximately unbiased *P*-values < 0.05, while dashed lines represent nonsignificant branches based on 20,000 bootstraps.

To test how the genes identified across the 85 genotypes may influence virulence across the phylogenetic relationship of the hosts, we assigned the candidate genes to the phylogenic tree of the hosts. This representation of the evolutionary history of the hosts revealed that genus level (e.g. Cichorium Fig. 6a) and order (e.g. Brassicales, Fig. 6a) shared more genes than the clade level (e.g. Rosids and Asterids, Fig. 6a). However, given the complex patterns of shared genes across the hosts (Fig. 5a), this analysis only accounts for 83 genes associated with multiple hosts that share a phylogenetic relationship.

As an alternative analysis, we used the candidate genes to test if the similarity in candidate virulence genes associated with the host species may show relationships that do not track the evolutionary phylogenetic relationships of the hosts. This test was done using a hierarchical clustering of the candidate genes effect across the different hosts (Fig. 6b). Based on the fungal genetic variance, there is a significant discord with the plant phylogenetic relationship. Specifically, Botrytis virulence variation connects several Asterid-Rosid pairs including chicory (Asterids) clustering with soybean (Rosids) and sunflower (Asterids) clustering with turnip (Rosids) (Fig. 6b). Similarly, tomato and endive identify a common set of 80 Botrytis candidate virulence genes (Fig. 6b) that are functionally diverse. These associations suggest that either Botrytis growth is opportunistic or it may use similar virulence strategies across relatively unrelated host species. Such an observation might relate to the recognition of phytochemicals (Kuroyanagi *et al*. 2022) and the activation of detoxification mechanisms that can be effective on phytochemicals from distantly related species (Bulasag *et al*. 2023; Wu *et al*. 2024). Plant defense phytochemicals often show convergent evolution in distantly related hosts like glucosinolates present in both the Brassicales and unrelated Drypetes (Pichersky and Lewinsohn 2011; Negin and Jander 2023). However, how Botrytis ability to develop lesions on diverse hosts is facilitated by its genetic landscape remains to be fully discovered.

### Functionally diverse genes contribute to the lesion area across hosts

**Fig. 7.**
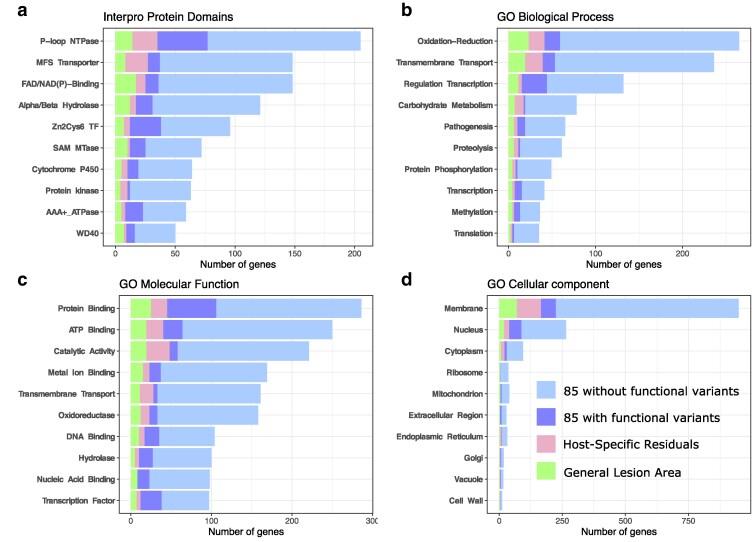
The Botrytis genes associated with the lesion area by GWAS cover a large range of functions. This figure provides an overview of the most frequent annotations across the 3 datasets. The count of Botrytis genes identified in the 85 genotypes dataset is in blue with partitioning of genes without and with functional effect SNPs. The count of Botrytis genes identified in the host-specific residuals dataset is overlaid in red. The count of the Botrytis genes identified in the general lesion area dataset is overlaid in green. a) Number of genes for the top 10 protein domains as identified by Interpro. b) Number of genes for the top 10 gene ontologies for biological process. c) Number of genes for the top 10 gene ontologies for molecular function. d) Number of genes for the top 10 gene ontologies for cellular components.

To assess the molecular strategies deployed by Botrytis, we analyzed the genomic annotations of the GWAS-identified genes and reported the top 10 categories (Fig. 7). The ranking of the top functions was consistent across the 85 genotypes, the general lesion area, and the host-specific residuals suggesting that the general and host-specific genetics behind the lesion area are drawing from similar mechanisms. The genes associated with lesion area across datasets come from diverse biological processes (Fig. 7b), molecular functions (Fig. 7c), and cellular components (Fig. 7d). The top annotations reflect biological processes (Fig. 7b) such as oxidative stress responses (265 genes), transmembrane transport (236 genes), regulation of the transcription (132 genes), and biosynthetic and catabolic processes that are known for their role in the interaction with the host. The key role of transmembrane transport (Fig. 7b) is driven by 148 MFS transporters (Fig. 7a), including 37 genes with functional effect variants. The role of regulation of the transcription (Fig. 7d) is driven by the 96 Zn(2)-C6 transcription factors (Fig. 7c), including 38 genes with functional effect variants. The role of biosynthetic and catabolic processes (Fig. 7d) is also associated with the 64 cytochrome P450 (Fig. 7c), including 19 genes with functional effect variants.

## Discussion

This study investigated the genetic architecture of Botrytis virulence as measured by the lesion area on the leaves of 85 plant genotypes from 8 hosts. Using these data, we estimated the contributions of Botrytis lesion development specific to each host and general across all hosts. All 3 trait sets found similar genetic architecture with candidate loci being evenly distributed across the 16 core chromosomes without evidence of virulence-enriched genome areas and loci of small effect sizes. This genome-wide distribution is consistent with previous genome scans for evolutionary selection (Van Kan *et al*. 2017; Atwell *et al*. 2018; Mercier *et al*. 2021), which did not detect hotspots for variation. The absence of the candidate SNPs and genes on the accessory chromosomes is consistent with their previously documented low gene content (<20) (Van Kan *et al*. 2017) and low polymorphism (Atwell *et al*. 2018). Furthermore, the genes on the accessory chromosomes are expressed less frequently in planta than genes on the core chromosomes (Zhang *et al*. 2019). This genetic architecture of virulence contrasts with other plant pathogens that have physically delineated genetic architectures such as the 2-speed genome model (Dong *et al*. 2015). In other fungal species, accessory chromosomes contain host-specific virulence factors and toxin biosynthesis gene clusters that have enriched expression in planta (Feurtey *et al*. 2020). While current data do not support a major role in the host range for accessory chromosomes in Botrytis, further work with high-quality long-read genomes is needed to identify the full diversity of these chromosomes in the species. The accessory chromosomes in Botrytis could be reservoirs of TEs contributing to small RNA production rather than virulence genes (Simon *et al*. 2022).

Given the generalist nature of *B. cinerea*, we examined how the genetic architecture partitioned general lesion formation across hosts vs host-specific effects. The candidate genes revealed similar mechanisms and gene sets across the 3 datasets. This suggests that while it is possible to identify contributions that generally alter all host interactions or that are host-specific, these associate with similar genes and mechanisms. Furthermore, candidate genes associated with both host-specific and general lesions development are largely shared with other Botrytis species (Fig. 4; Supplementary Fig. 11), including species that specialize in infecting monocot plants (Valero-Jiménez *et al*. 2019, 2020). This suggests that virulence and host range variation in *B. cinerea* may not primarily rely on the emergence of new genes or mechanisms, but rather on alleles that adjust existing ones. An alternative hypothesis could be that Botrytis is largely a saprotroph with opportunistic pathogenesis with the SNPs being driven by neutral processes rather than any host specialization. Counter to this is the observation that *B. cinerea* has a number of defense mechanisms that are distinct for host-specific defense compounds like glucosinolates, camalexin, and tomatine (Stefanato *et al*. 2009; Buxdorf *et al*. 2013; You *et al*. 2024). Similarly, nonsynonymous variation within the PG1 enzyme was associated with the differential ability to degrade pectin from distinct lineages (Rowe and Kliebenstein 2007). As such, there is evidence for host-specific mechanisms within *B. cinerea* (Mercier *et al*. 2021). A model of generalist adaptation by adjustment of existing genes and mechanisms rather than novel gene evolution is also emerging in *S. sclerotiorum.* This species closely related to Botrytis has consistent transcriptional variation across different hosts (Allan *et al*. 2019; Kusch *et al*. 2021).

Conducting GWAS on leaf lesions from 8 species showed that the majority of candidate genes do not track the host phylogeny (Fig. 6). This raises the possibility that the variation in these genes may not respond specifically to the hosts’ evolutionary changes but instead may function as a form of bet-hedging (de Jong *et al*. 2011; Haaland *et al*. 2020). The adjustment of sets or networks of Botrytis genes could be a solution to fluctuations in host diversity. In locations with long-term stable agricultural monocropping, Botrytis may have the opportunity to adapt genes/mechanisms to better infect specific hosts or plant families. A modest signal for host specialization was documented in grapevine and tomatoes with the signature of positive selection on cell wall degradation enzymes and oxidative stress genes (Mercier *et al*. 2021). Future studies will need to assess if the Botrytis genes associated with general vs host-specific lesion differential respond in environments that vary in host diversity from monocropping systems to natural environments.

One complication influencing all Botrytis studies is the conditionality of this host–pathogen interaction. The lesion formation is dependent on the genetics of the host and the pathogen as well as the infected tissue and the environment in which the interaction is occurring. In an attempt to address this within this study, we included a wide range of host genotypes both across and within host species. We also conducted independent experiments separated across seasons. We finally chose to focus on a single tissue, the leaf, across all the hosts to maximize comparability and enhance our ability to conduct a large-scale experiment. Because of this focus, the genes identified in this study represent a minimum set of candidate genes, as there are likely other genes influencing interactions in different tissues or under untested environmental conditions. By including a diverse range of hosts, our general observations about a highly polygenic architecture will likely hold true in other systems and environments. However, future experiments will need to test this across a diversity of tissues, environments, field conditions, and whole plants.

This work parsing Botrytis virulence across hosts between general and host-specific measures suggests that to successfully infect thousands of plants (Elad *et al*. 2016; Singh *et al*. 2023b), *B. cinerea* likely relies on a large portion of its genome involved in a multilayer quantitative system. Some virulence mechanisms might be host-specific while others might contribute to Botrytis virulence and potentially growth across multiple hosts, potentially unrelated. This complex quantitative system is also highly redundant as shown by the resilience of phytotoxic machinery to serial knockouts (Leisen *et al*. 2022) and the 4 different strategies for the detoxification of a single phytoalexin (You *et al*. 2024). To precisely map how Botrytis recognizes the hosts, how the hosts impact the virulence strategies, and how the natural genetic diversity of Botrytis strains contributes to the virulence and host range will require a massive transcriptome sequencing effort and validation of gene networks rather than individual genes.

## Supplementary Material

iyaf079_Supplementary_Data

## Data Availability

Correspondence and requests for materials should be addressed to kliebenstein@ucdavis.edu. The datasets and R codes are available on Dryad: https://doi.org/10.5061/dryad.j6q573npm. Supplemental material available at GENETICS online.
