# Supplementary material for: Polygenic strategies for host-specific and general virulence of Botrytis cinerea across diverse eudicot hosts
Source: Genetics. 2025 Jun 9;230(3):iyaf079. doi: 10.1093/genetics/iyaf079 (PMC12239214; doi:10.1093/genetics/iyaf079)
Supplement: iyaf079_Supplementary_Data [file iyaf079_supplementary_data.zip › Figure_S10_GENETICS-2025-308097.pdf]

A)

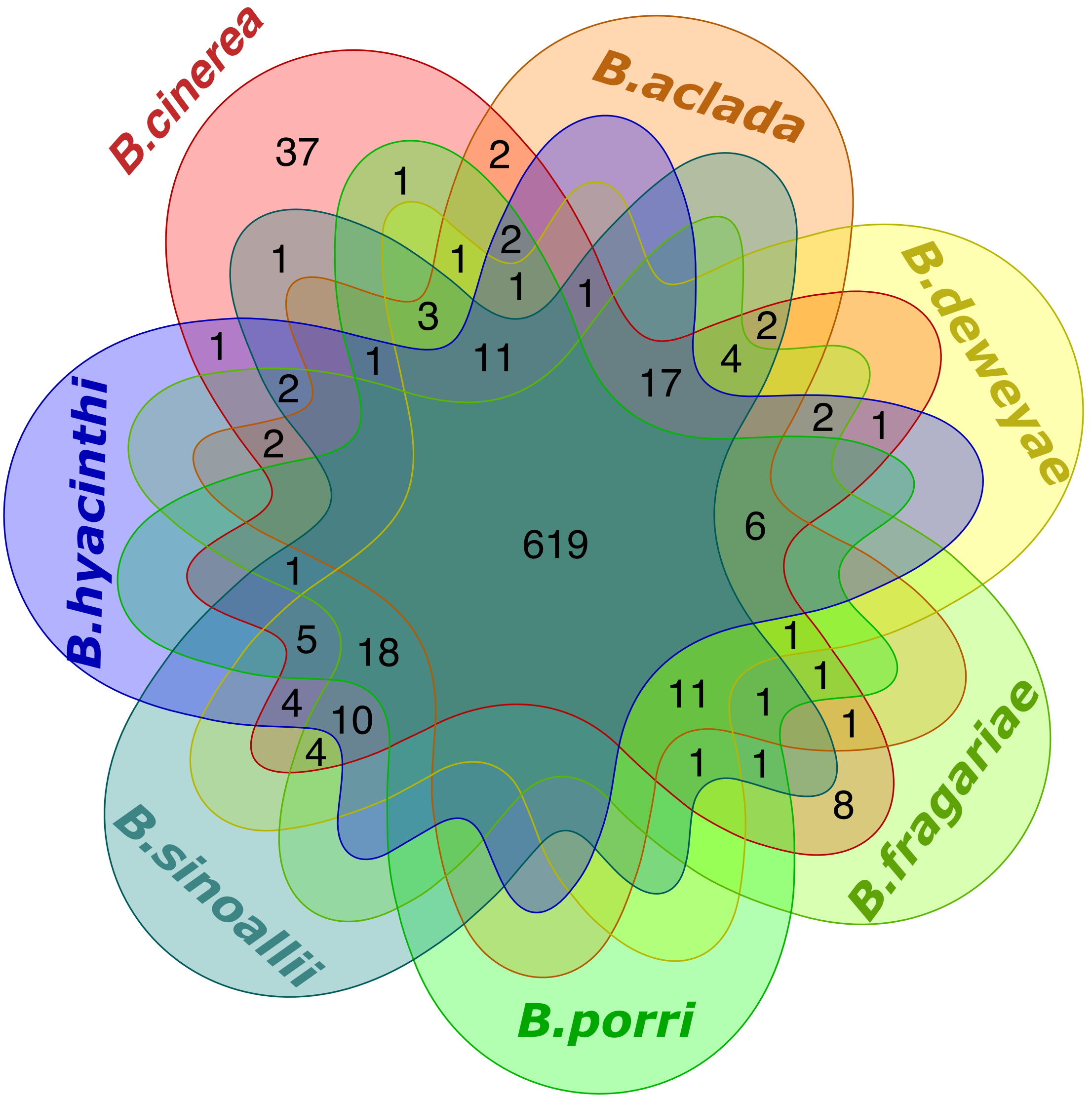
B.cinerea
B.aclada
B.deweyae
B.fragariae
B.porri
B.sinoallii
B.hyacinthi
37
2
1
2
1
1
1
1
3
1
1
2
2
1
11
17
2
4
2
1
619
6
1
5
18
4
10
4
1
1
11
1
1
1
1
8

B)

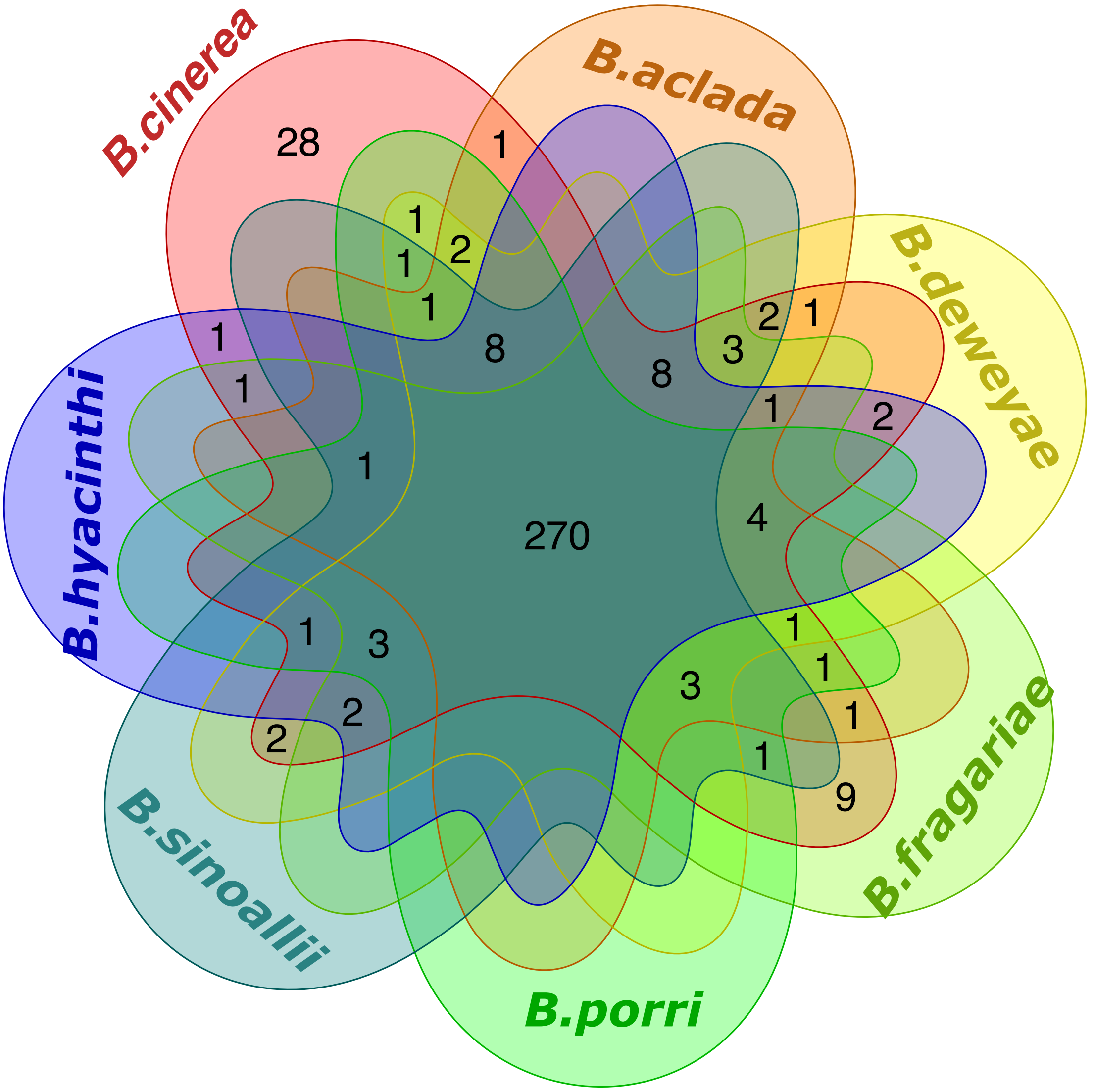
B.cinerea
B.aclada
B.deweyae
B.fragariae
B.porri
B.sinoallii
B.hyacinthi
28
1
1
2
1
1
1
1
8
8
2
1
3
1
2
1
270
4
1
3
2
2
1
1
3
1
1
9
