# Supplementary material for: Polygenic strategies for host-specific and general virulence of Botrytis cinerea across diverse eudicot hosts
Source: Genetics. 2025 Jun 9;230(3):iyaf079. doi: 10.1093/genetics/iyaf079 (PMC12239214; doi:10.1093/genetics/iyaf079)
Supplement: iyaf079_Supplementary_Data [file iyaf079_supplementary_data.zip › Supplemental_Material_Legends_GENETICS-2025-308097.docx]

**Supplemental Tables and Figures Legends**

**Table S1:** **Information for the 85 host genotypes.** The accession ID corresponds to the germplasm. Information on the species and subspecies, Taxon, order, clade, improvement status, cultivar name, geographical origin are provided when available. Abbreviations: USDA Grin (the U.S. Department of Agriculture Germplasm Resources Information Network), UK vegetable genebank (Warwick University), TGRC (UC Davis Tomato Genetic Resource Center), ABRC (Arabidopsis Biological Resource Center).

**Table S2: Description of SNPs associated to lesion area across the 85 genotypes.** Columns A-D include the chromosomes and positions. Columns E-M contain annotations, including the genomic features and the functional effects. Gene500 consider SNPs within 500bp of the start or end of the gene when Within_genes consider only SNPs between start to end of the gene. Columns N-W describe significant associations (coded by 1) to lesion area. Columns V and W provide respectively the number of genotypes and species the SNP was associated to.

**Table S3: Description of SNPs associated to the host-specific residuals.** Columns A-D include the chromosomes and positions. Columns E-M contain annotations, including the genomic features and the functional effects. Gene500 consider SNPs within 500bp of the start or end of the gene when Within_genes consider only SNPs between start to end of the gene. Columns N-U describe significant associations (coded by 1) to lesion area. Column U provides the number of species the SNP was associated to.

**Table S4: Description of SNPs associated to the general lesion area.** Columns A-D include the chromosomes and positions. Columns E-M contain annotations, including the genomic features and the functional effects. Gene500 consider SNPs within 500bp of the start or end of the gene when Within_genes consider only SNPs between start to end of the gene.

**Table S5: Description of all candidate genes and non-coding RNAs identified by GWAS.** Significant associations are denoted by YES or coded by 1, when the absence of association is noted by NO or coded by 0. Columns B-K describe the 85 genotypes dataset. Column L describes whether the genes were associated to the general lesion area. Columns M-U describe the residual dataset. Columns V-AL describe the annotations with gene names, genomic location, protein features, and function prediction as present on fungidb.org in January 2024.Column AM provides whether the genes were functionally validated with the reference. Column AN contains manual annotations such as whether the gene was detected in the secretome or surfactome.

**Figure S1: Estimation of the general and host-specific contributions to the lesion area.** Correlation of the general lesion area (x-axis) and the host-specific lesion area (y-axis) across the 96 Botrytis strains (individual dots). The blue lines represent the linear regression with the confidence interval in grey. The host-specific residuals were calculated from those linear regressions.

**Figure S2: Permutation-based significance thresholds for the posterior inclusion probability (PIP).** For the lesions on each species (phenotype), the significance thresholds were estimated by running the BSLMM model with ten random permutations of SNPs positions. PIP values larger than 1.7 x10^-4^ are equivalent to 5% chance of a false positive, while 3.4 x10^-4^ and 4.2 x10^-4^ are respectively at 1% and 0.5% thresholds.

**Figure S3: Overview of the phenotypic data (Caseys *et al.*, 2021) used to conduct the GWAS.** A) Genetic heritability of the lesion area of 96 strains of Botrytis on 85 plant genotypes colored by host species. The heritability was calculated as the percentage of variance in lesion area explained by Botrytis strains. B) Variation in lesion area at 72 hours post inoculation across the 96 Botrytis strains for each plant genotype.

**Figure S4: Genetic architecture of Botrytis lesion area on 85 genotypes** For each BSLMM hyperparameter, the mean of the recorded iterations for the 20 separate BSLMM runs is provided. The plant accessions on which the lesion area was measured (phenotypes) are colored by species. A) Rho describes the proportion of genome-wide small effects (rho=0 polygenic) to relatively larger effects (rho=1 oligogenic). B) Total percentage of phenotypic variance explained (PVE) by all SNPs included in the model. C) Number of SNPs with relatively larger effects estimated by the oligogenic model. D) Percentage of the PVE explained by the SNPs with relatively larger effects.

**Figure S5: Genetic architecture of the general lesion area across all hosts and the host-specific residuals.** For each BSLMM hyperparameter, the mean of the recorded iterations for the 20 separate BSLMM runs is provided. The plant accessions on which the lesion area was measured (phenotypes) are colored by species. A) Rho describes the proportion of genome-wide small effects (rho=0 polygenic) to relatively larger effects (rho=1 oligogenic). B) Total percentage of phenotypic variance explained (PVE) by all SNPs included in the model. C) Number of SNPs with relatively larger effects estimated by the oligogenic model. D) Percentage of the PVE explained by the SNPs with relatively larger effects.

**Figure S6: SNPs significantly associated (LFSR<0.05) to the lesion area on 85 genotypes** **show no enrichment for genomic features.** Dots are the count SNPs detected by GWAS categorized based on their genomic locations (UTR, CDS, Intron, Intergenic, TE) and functional effects. The grey zones represent the minimum and maximum range observed in the thousand random SNP sets. The figure on the right is a zoom up to maximum 5000 SNPs of the figure on the left.

**Figure S7: Manhattan plot of the effect size of SNPs significantly associated (PIP>1.7x10^-4^) to hosts-specific residuals.** The x-axis represents positions along the 16 core chromosomes. The y-axis is the effect size [cm^2^] as estimated by the BSLMM. The dot colors indicate the host the SNP was associated to.

**Figure S8: Hierarchical clustering of SNPs significantly associated (LFSR<0.05) to the lesion area on 85 host genotypes.** Branch length represents the correlation distances between genotypes based on the significance status of each SNP on each genotype. Dashed lines represent non-significant (p-val>0.05) branches from bootstrap resampling. The genotype names are colored based on the host species.

**Figure S9: Hierarchical clustering of SNPs associated (PIP>1.7x10^-4^) to the host-specific residuals.** Branch length represents the correlation distances between hosts based on the significance status of each SNP on each species. Full lines represent significant (p-val<0.05) branches from bootstrap resampling.

**Figure S10. The *B.cinerea* genes detected by GWAS across (A) the host-specific residuals and (B) the general lesion area are highly syntenic with other Botrytis species.** Venn diagram with the count of *B. cinerea* B05.10 (clade 1, polyphagy index=54.1) genes shared with *B. fragariae* (clade 1, polyphagy index=1), *B. aclada* (clade 2, polyphagy index=1.4), *B. deweyae* (clade 2, polyphagy index=1), *B. porri* (clade 2, polyphagy index=1.4), *B. hyacinthi* (clade 2, polyphagy index=3.2) and *B. sinoallii* (clade 2, polyphagy index=1.4).

**Figure S11: Synteny among seven Botrytis species.** A) Venn diagram with the count of orthologs between *B.cinerea* (B05.10 reference genome) and six other Botrytis species. B) Number of candidate genes identified by GWAS (dots) with no (identified only in *B.cinerea*), partial (identified in 2 to Botrytis 6 species), or full synteny (identified in 7 Botrytis species). The grey zones represent the minimum and maximum range observed in the thousand random gene sets. C) Number of candidate genes identified by GWAS (dots) with orthologs in the Botrytis species. The grey zones represent the minimum and maximum range observed in the thousand random gene sets.
