# Supplementary figures and images for: Polygenic strategies for host-specific and general virulence of Botrytis cinerea across diverse eudicot hosts
Source: Genetics. 2025 Jun 9;230(3):iyaf079. doi: 10.1093/genetics/iyaf079 (PMC12239214; doi:10.1093/genetics/iyaf079)

A)

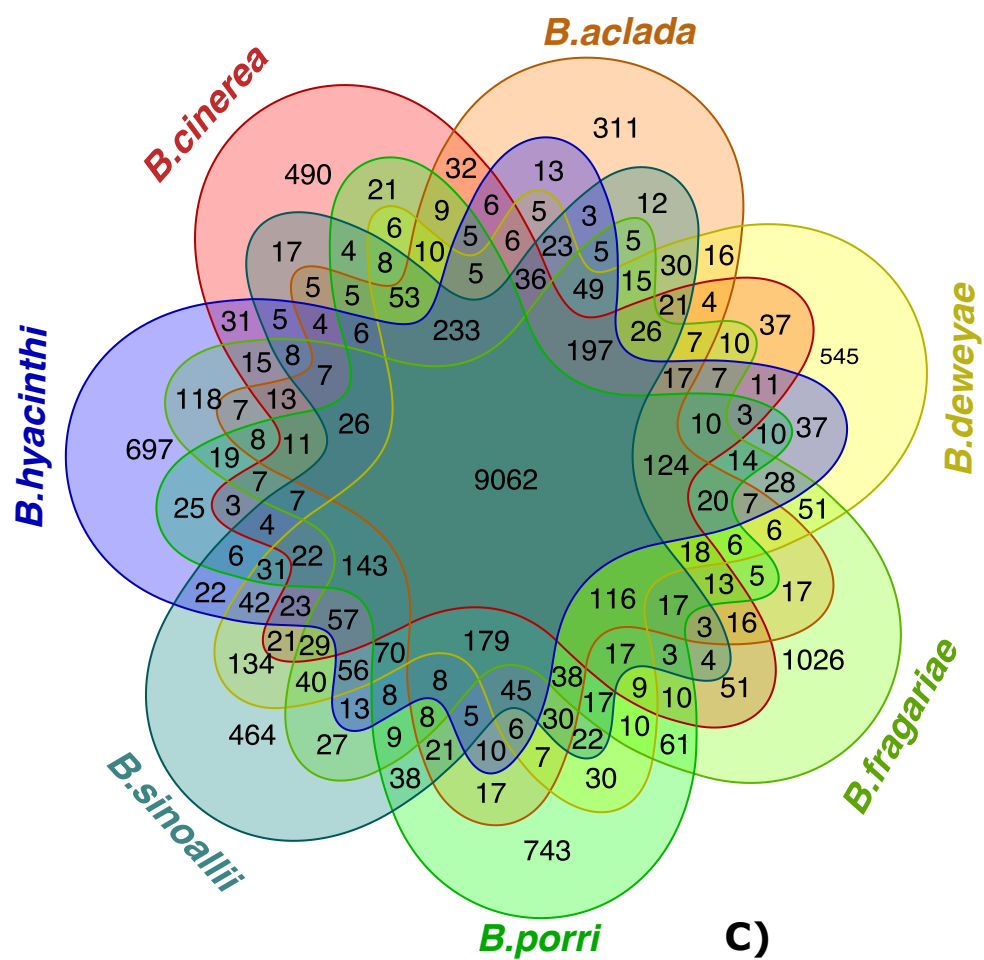

B)

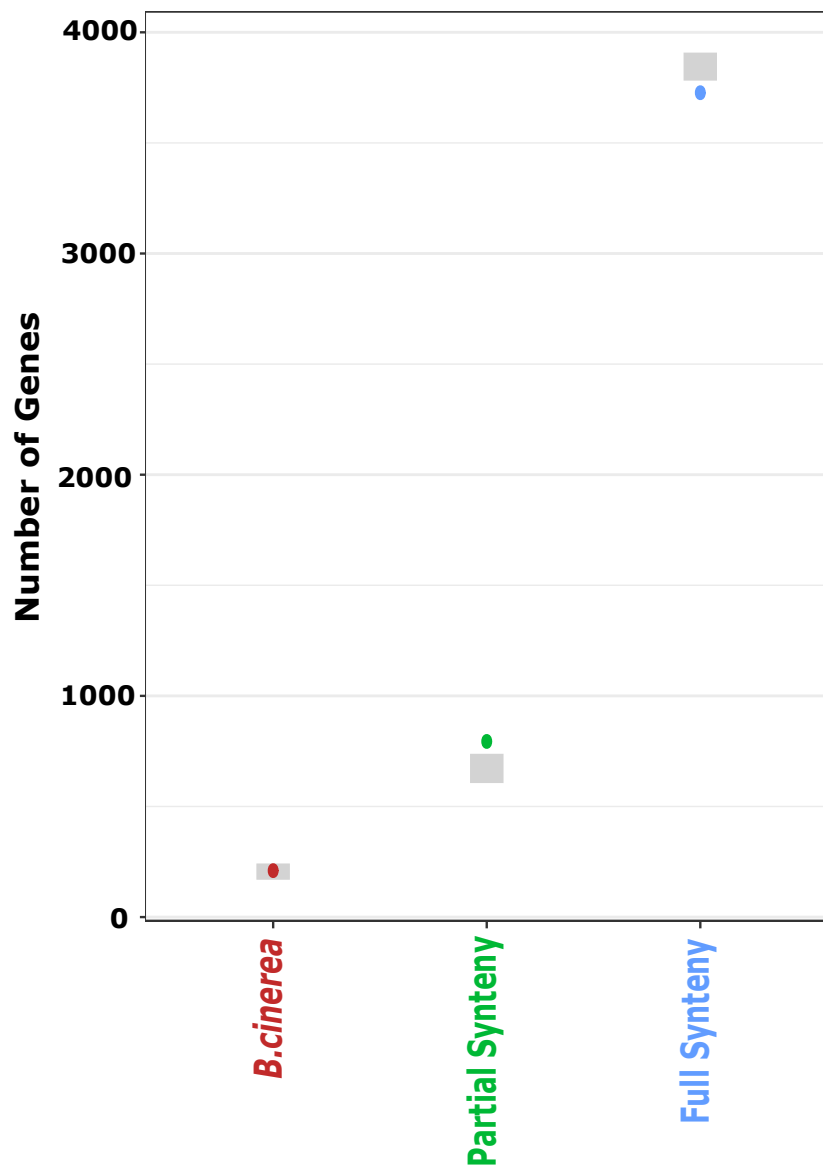

C)

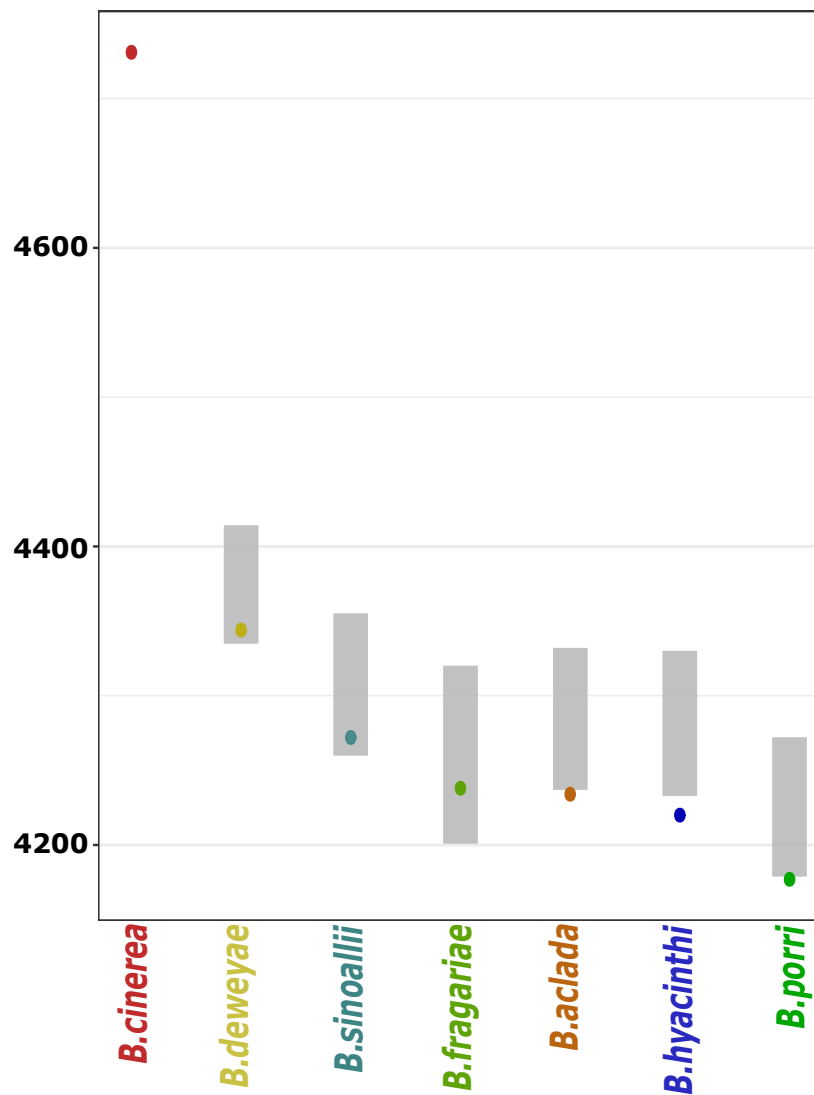

Supplement: iyaf079_Supplementary_Data [file iyaf079_supplementary_data.zip › Figure_S11_GENETICS-2025-308097.pdf]

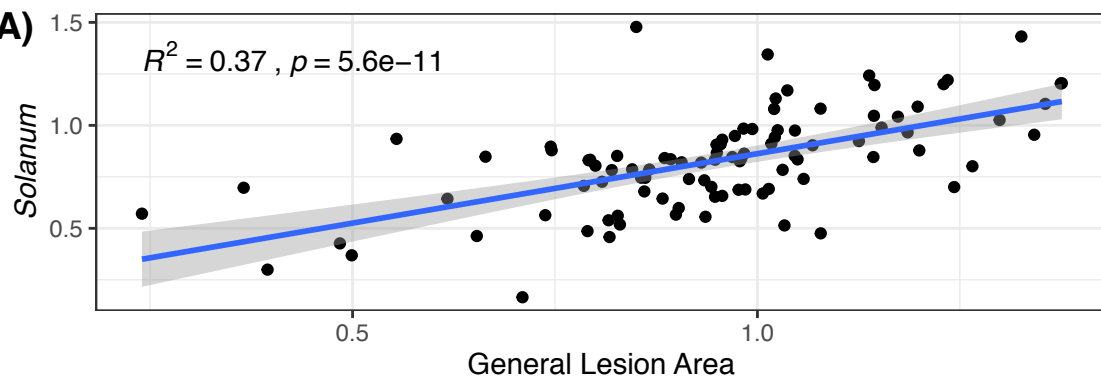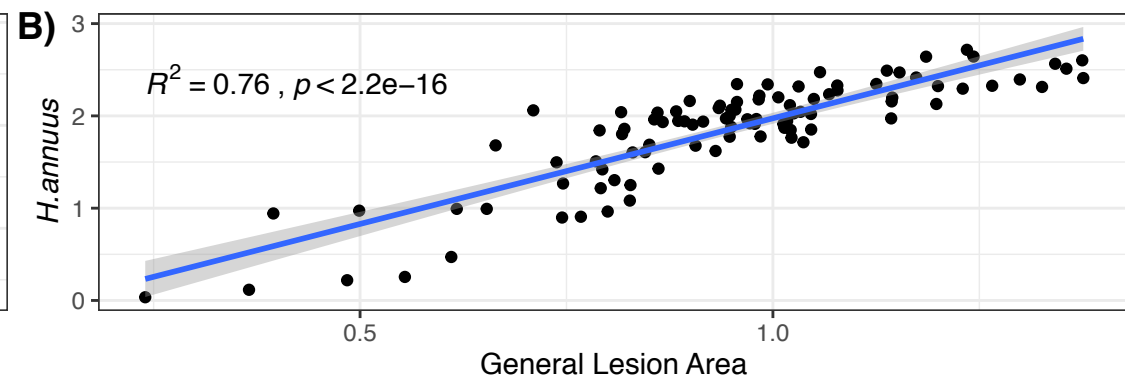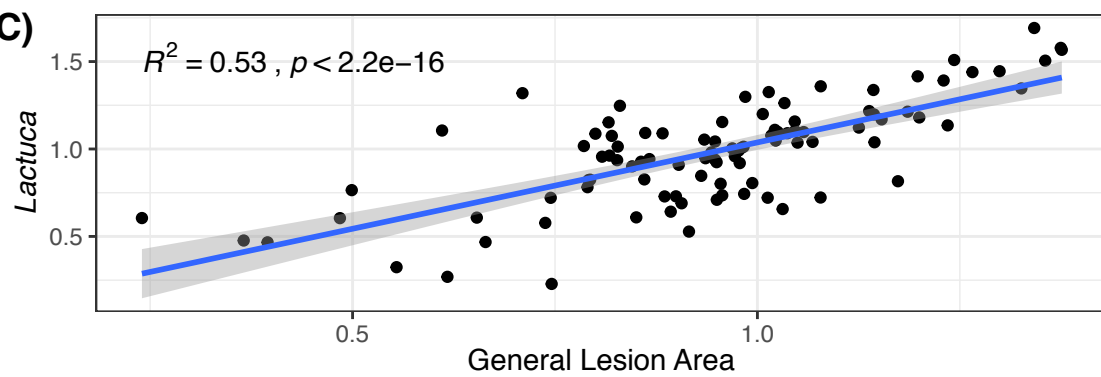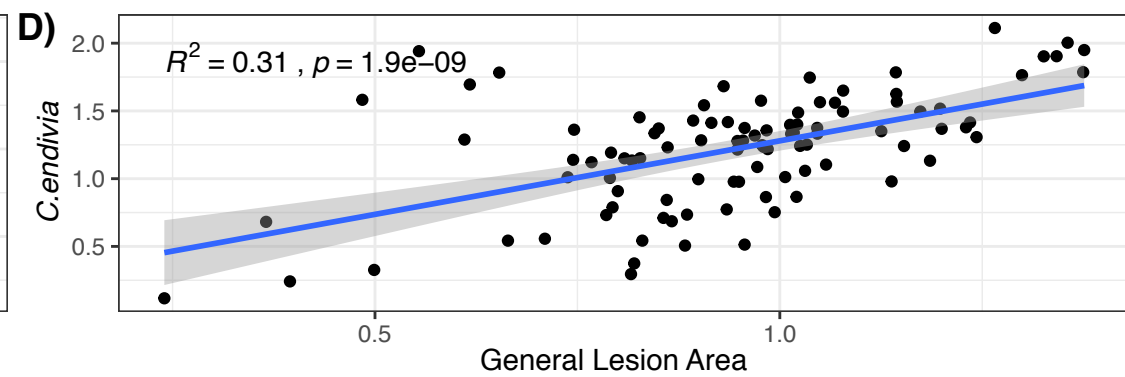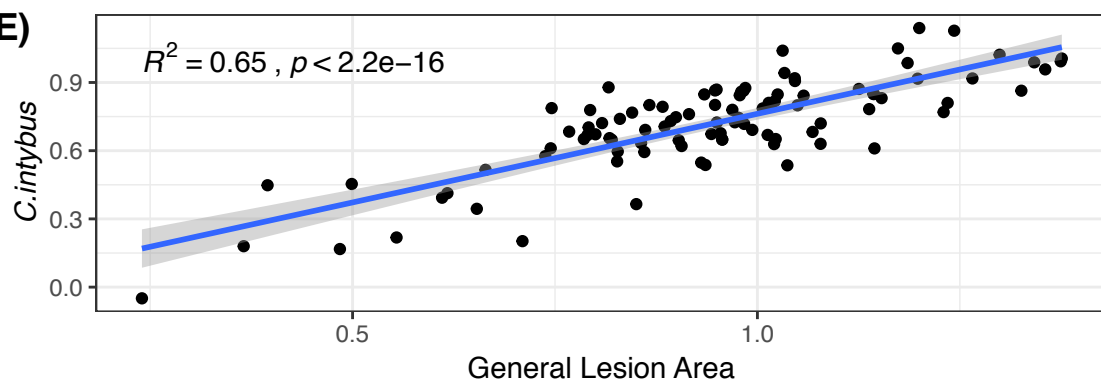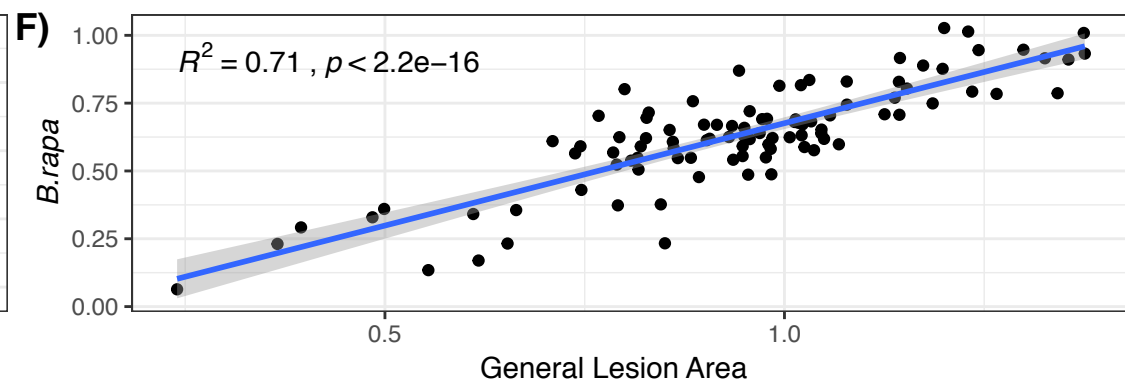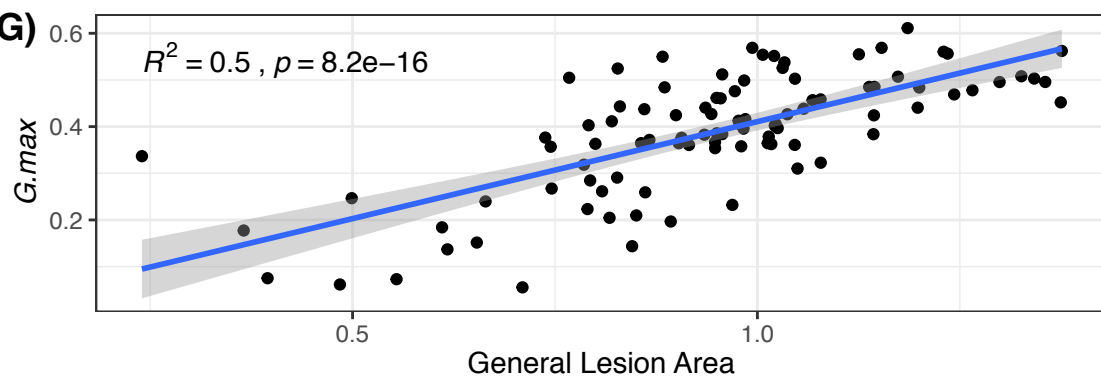

Supplement: iyaf079_Supplementary_Data [file iyaf079_supplementary_data.zip › Figure_S1_GENETICS-2025-308097.pdf]

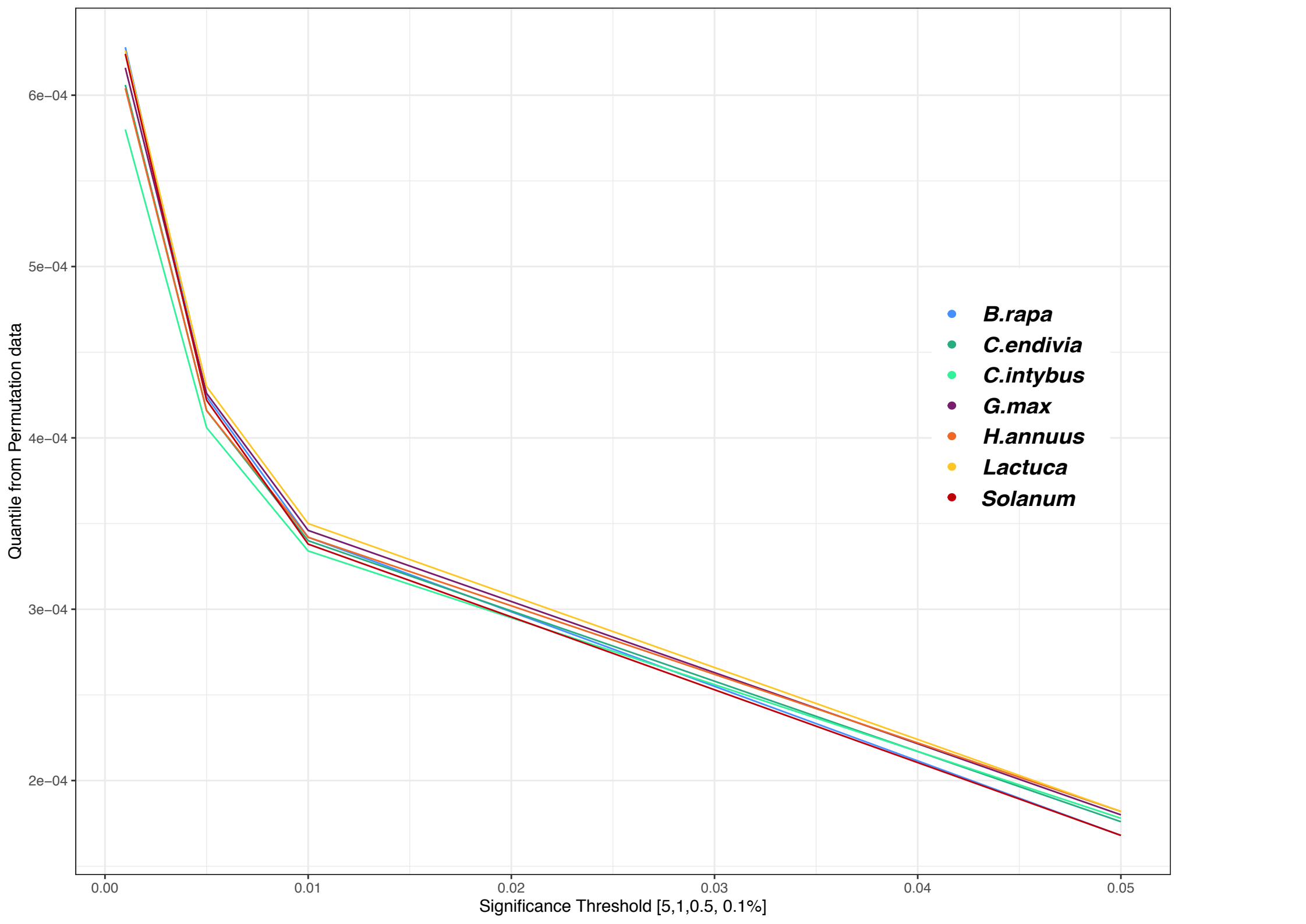

Supplement: iyaf079_Supplementary_Data [file iyaf079_supplementary_data.zip › Figure_S2_GENETICS-2025-308097.pdf]

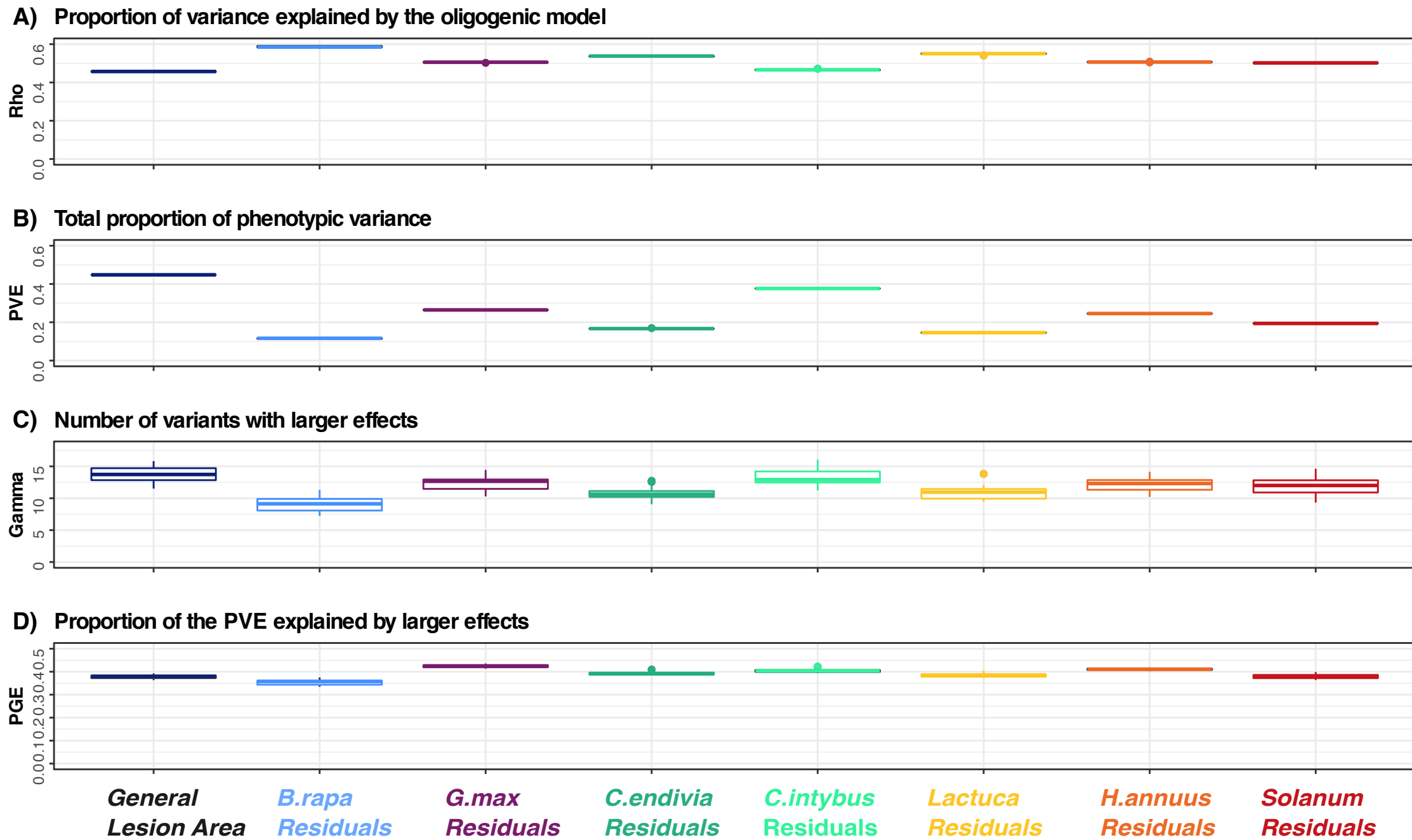

Supplement: iyaf079_Supplementary_Data [file iyaf079_supplementary_data.zip › Figure_S5_GENETICS-2025-308097.pdf]

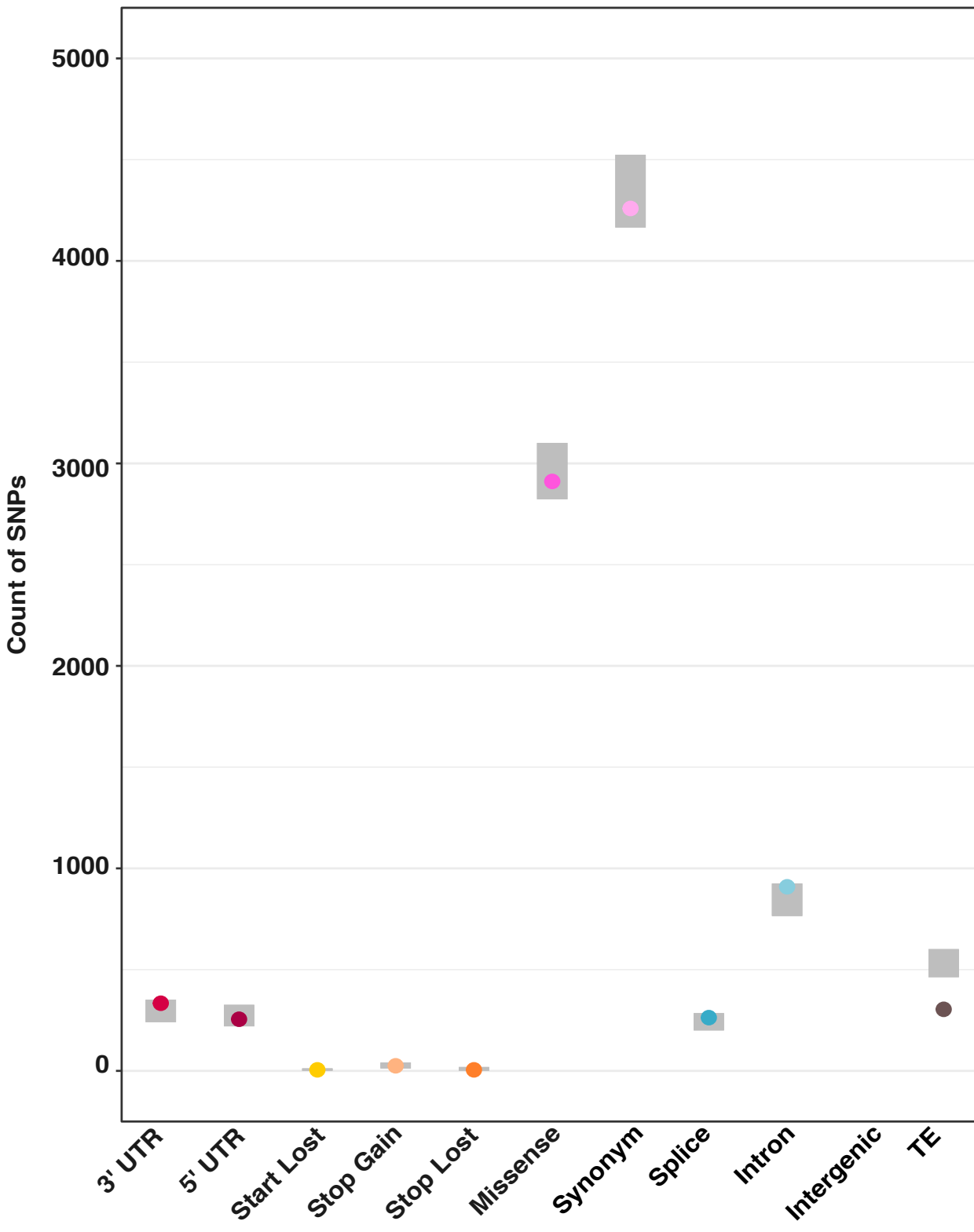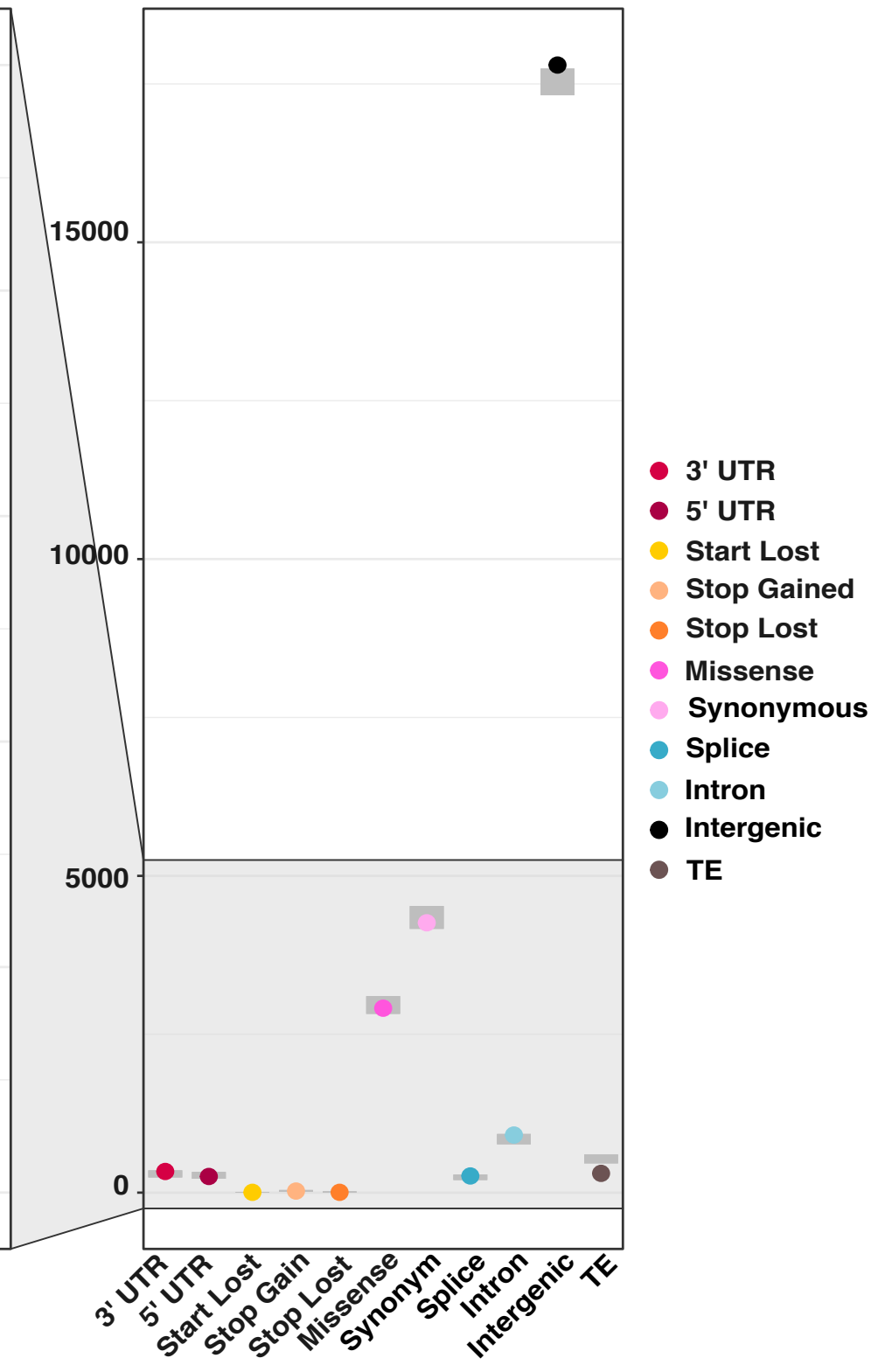

Supplement: iyaf079_Supplementary_Data [file iyaf079_supplementary_data.zip › Figure_S6_GENETICS-2025-308097.pdf]

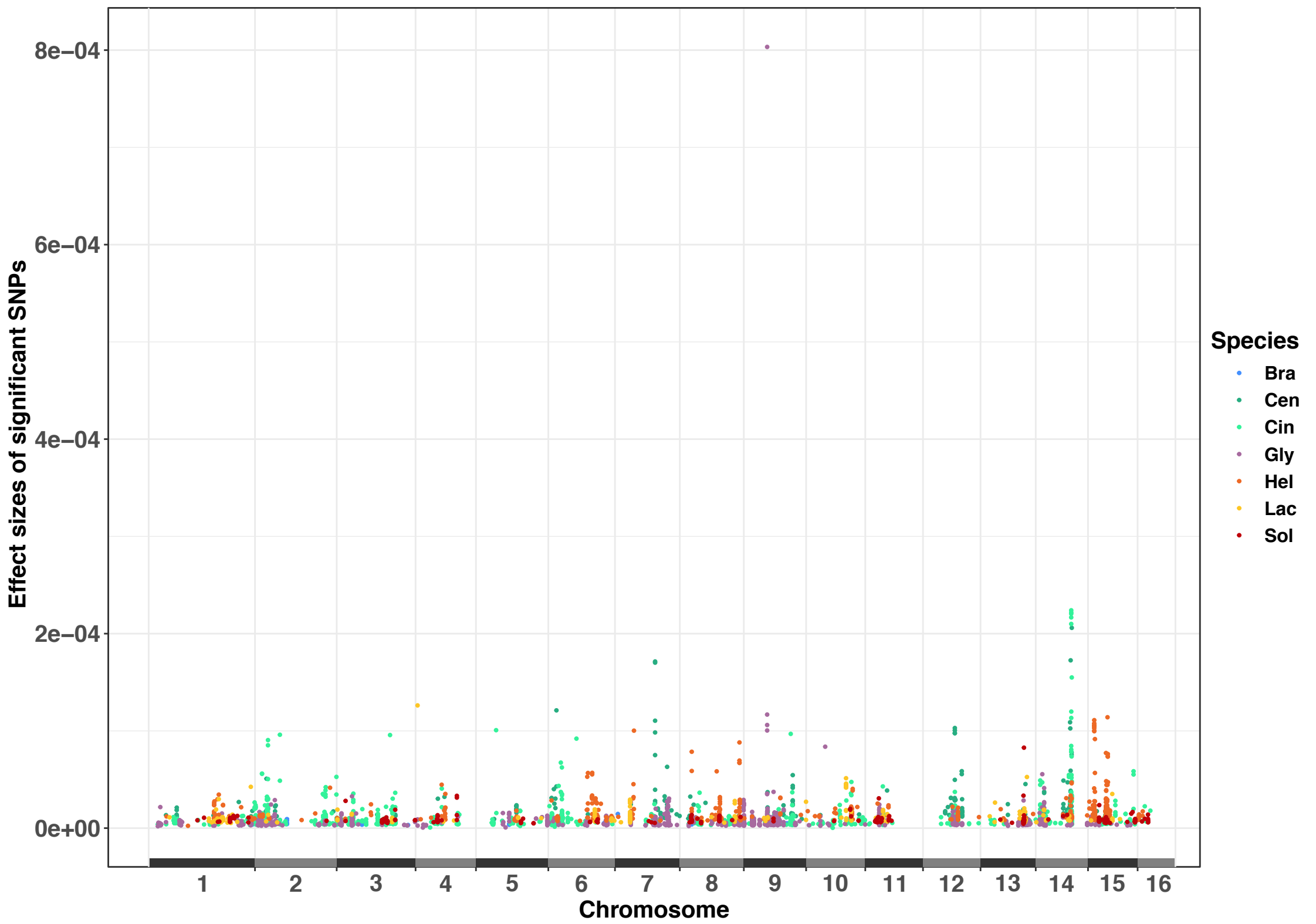

Supplement: iyaf079_Supplementary_Data [file iyaf079_supplementary_data.zip › Figure_S7_GENETICS-2025-308097.pdf]

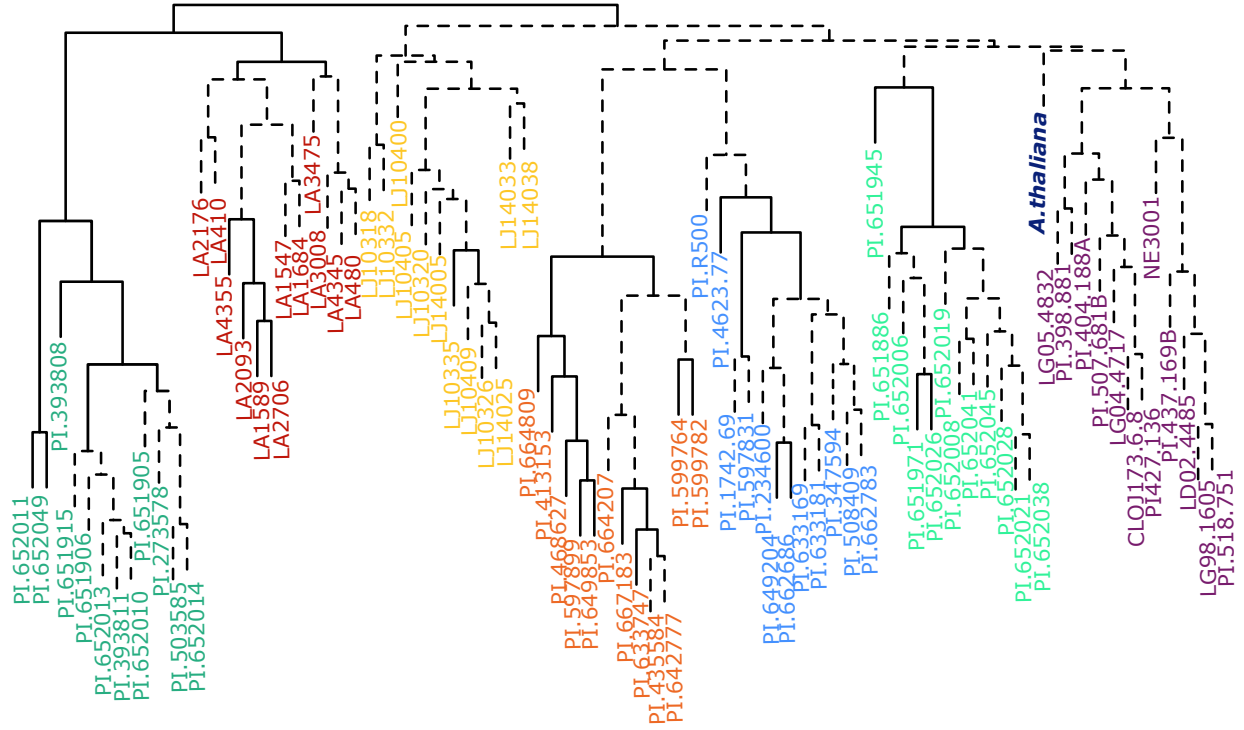

*C. endivia*

*Solanum*

*Lactuca*

*H. annuus*

*B. rapa*

*C. intybus*

*G. max*

Supplement: iyaf079_Supplementary_Data [file iyaf079_supplementary_data.zip › Figure_S8_GENETICS-2025-308097.pdf]

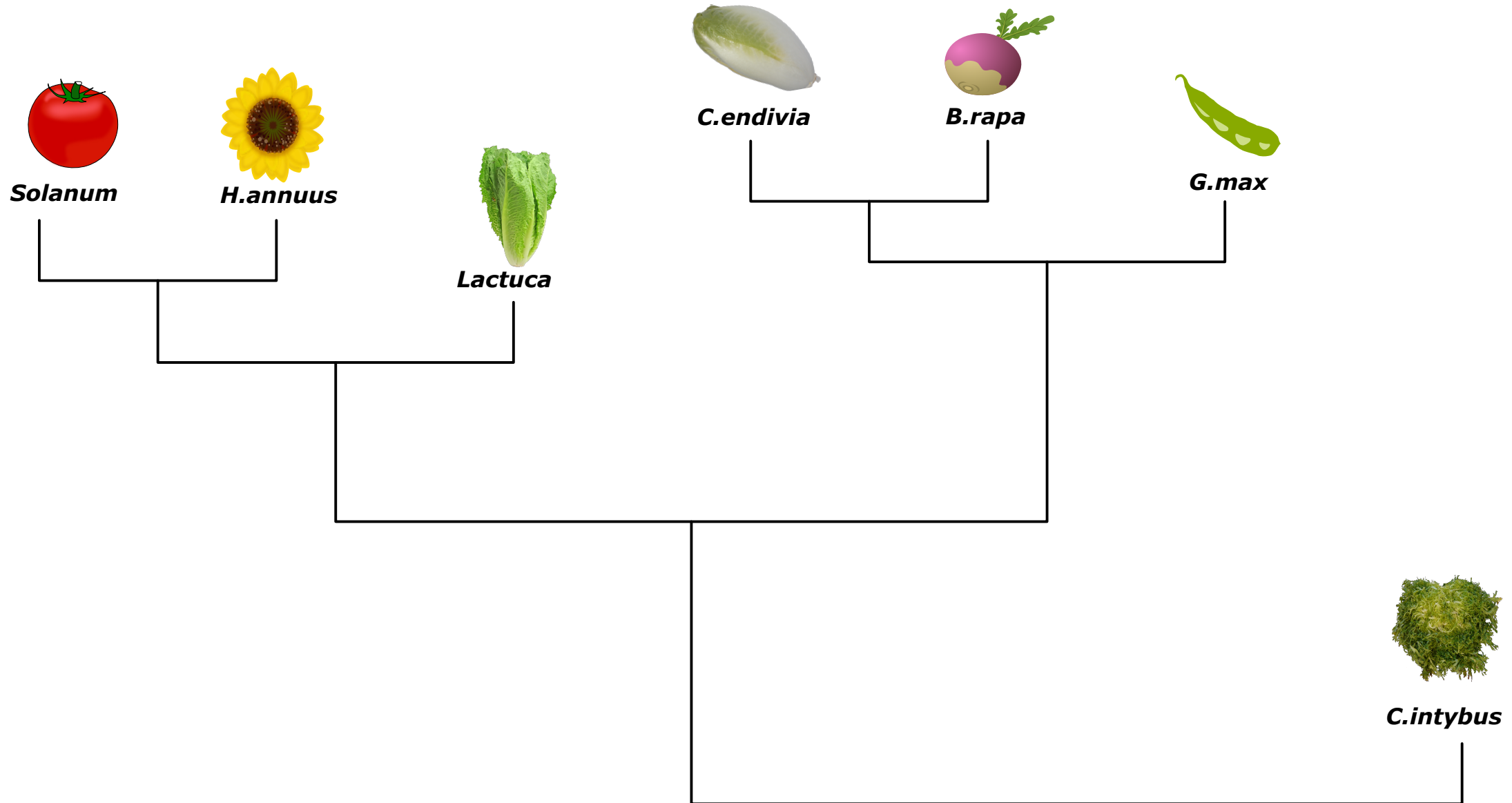

Supplement: iyaf079_Supplementary_Data [file iyaf079_supplementary_data.zip › Figure_S9_GENETICS-2025-308097.pdf]
