# Supplementary material for: Polygenic strategies for host-specific and general virulence of Botrytis cinerea across diverse eudicot hosts
Source: Genetics. 2025 Jun 9;230(3):iyaf079. doi: 10.1093/genetics/iyaf079 (PMC12239214; doi:10.1093/genetics/iyaf079)
Supplement: iyaf079_Supplementary_Data [file Figure_S3_GENETICS-2025-308097.pdf › Figure_S3_GENETICS-2025-308097.pdf]

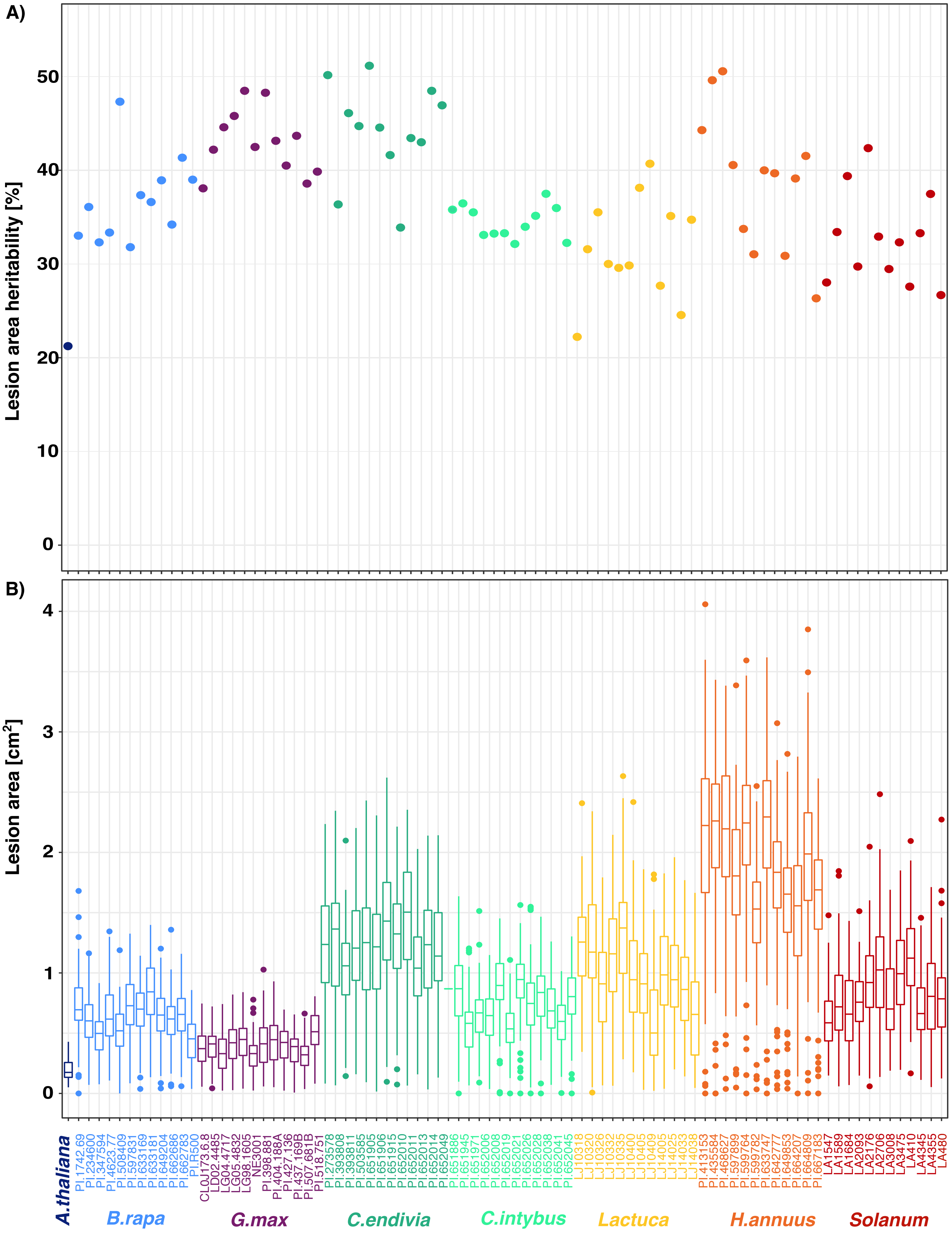

A)
Lesion area heritability [%]
0
10
20
30
40
50
B)
Lesion area [cm2]
0
1
2
3
4
A.thaliana
PI.1742.69
PI.234600
PI.347594
PI.4623.77
PI.508409
PI.597831
PI.633169
PI.633181
PI.649204
PI.662686
PI.662783
PI.R500
CL0J173.6.8
LD02.4485
LG04.4717
LG05.4832
LG98.1605
NE3001
PI.398.881
PI.404.188A
PI.427.136
PI.437.169B
PI.507.681B
PI.518.751
PI.273578
PI.393808
PI.393811
PI.503585
PI.651906
PI.651915
PI.652010
PI.652011
PI.652013
PI.652014
PI.652049
PI.651886
PI.651945
PI.651971
PI.652006
PI.652008
PI.652019
PI.652021
PI.652026
PI.652028
PI.652038
PI.652041
PI.652045
LJ10318
LJ10320
LJ10326
LJ10332
LJ10335
LJ10400
LJ10405
LJ10409
LJ14005
LJ14025
LJ14033
LJ14038
PI.413153
PI.435584
PI.468627
PI.597899
PI.599764
PI.599782
PI.633747
PI.642777
PI.649853
PI.664207
PI.664809
PI.667183
LA1547
LA1589
LA1684
LA2093
LA2176
LA2706
LA3008
LA3475
LA410
LA4345
LA4355
LA480
B.rapa
G.max
C.endivia
C.intybus
Lactuca
H.annuus
Solanum
